# Comparing the Effectiveness of Digital 3D PDF vs. 3D-Printed Heart Models as Learning Aids for Echocardiography in Medical Students

**DOI:** 10.1007/s40670-025-02392-x

**Published:** 2025-04-29

**Authors:** Kunaal S. Sarnaik, Vishnu Ramasamy, Kelli Qua, Robert A. Jones, Susanne Wish-Baratz

**Affiliations:** 1 Case Western Reserve University School of Medicine, 10900 Euclid Avenue, Cleveland, OH 44106 USA; 2 Department of Materials Science and Engineering, Case Western Reserve University, Cleveland, OH USA; 3 Center for Medical Education, Case Western Reserve University School of Medicine, Cleveland, OH USA; 4 Department of Emergency Medicine, MetroHealth Medical Center, Cleveland, OH USA; 5 Department of Anatomy, Case Western Reserve University School of Medicine, Cleveland, OH USA

**Keywords:** Echocardiography, Three-dimensional technology, Medical education

## Abstract

**Background:**

Reconciling echocardiographic images with heart anatomy in sonographic cross-sections is challenging. We compared use of 3D-printed heart models to 3D Portable Document Format (PDFs) heart models as learning aids in understanding echocardiography.

**Methods:**

3D heart models were printed in five echocardiographic cross-sections: parasternal long axis, parasternal base short axis, parasternal ventricular short axis, apical four-chamber, and bicaval. Models were also reproduced as 3D PDFs on handheld tablets. Medical students were assigned to use 3D PDFs (control) or 3D-printed models (experimental) while performing transthoracic echocardiograms on standardized patients. Students were given immediate (after one session) and delayed (after four sessions in two months) post-tests measuring satisfaction, learning quality, and echocardiographic knowledge. Analysis was conducted using two-way analysis of variance (ANOVA) testing with a p-value threshold of 0.05, followed by post-hoc testing with adjustment for multiple comparisons when indicated.

**Results:**

One-hundred fifty students were surveyed. The experimental group reported greater satisfaction and learning quality relative to the control for the immediate and delayed periods (*p* < 0.01). Satisfaction and learning quality decreased in the control group from the immediate to the delayed period (*p* < 0.01); no difference was found in the experimental group. No difference in group echocardiographic knowledge scores was found with respect to the assigned 3D technology (*p* = 0.091). The control group’s knowledge score increased from the immediate to the delayed period (*p* < 0.01); no difference was found for the experimental group (*p* = 0.238).

**Conclusion:**

3D PDF and 3D-printed heart models achieve similar efficacy in supplementing student learning of echocardiography. However, students may prefer 3D-printed heart models.

**Supplementary Information:**

The online version contains supplementary material available at 10.1007/s40670-025-02392-x.

## Introduction

In recent years, there has been increased adoption of three-dimensional (3D) anatomical models in medical education as learning aids for understanding anatomical [1], imaging [2], and surgical principles [3]. With regard to learning of medical anatomy and ultrasound imaging specifically, 3D-printed models replicating structures such as the skull [4], spine [5, 6], heart [7–9], lungs [10], and larynx [11] have demonstrated efficacy in various classroom settings. Studies investigating use of such 3D-printed anatomical models suggest not only that these aids enhance learner understanding of anatomy and visuospatial orientation, but also that their integration with traditional methods such as anatomical atlases and cadaveric dissection may be superior to utilization of either strategy alone. Given their ease of production, these conclusions provide strong evidence behind readily adopting 3D-printed models into the medical education environment. However, despite these pedagogical advantages, maintenance of 3D-printed models remains labor-intensive and costly, particularly due to their susceptibility to material degradation over time due to suboptimal storage conditions and handling by instructors and learners [12]. Combined with the burdensome costs of 3D printer acquisition and polymer blends required to produce high-quality models, this precludes widespread integration of 3D-printed models into medical education environments despite demonstrated efficacy [13].

Given the continually rising global incidence and prevalence of cardiovascular disease [14], adequate understanding of echocardiography becomes increasingly important for medical providers each year [15]. Since mastery of echocardiography relies on reconciling heart anatomy and cardiovascular physiology in the context of the cardiac cycle, this easily accessible, widely utilized diagnostic modality also serves as a useful learning tool of basic cardiovascular concepts in preclinical medical student education [16]. However, learning of echocardiography is challenging given the variety of projections utilized, each depicting the heart in a distinct cross-section [15]. Specifically, students find it difficult to reconcile the two-dimensional (2D) cross-section depicted on the computer screen with 3D anatomy of the heart given the various orientations and projections employed [15]. The difficulty of learning echocardiography therefore presents itself as a suitable area of medical education where integration of 3D-printed anatomical heart models as supplemental learning aids may be useful. In fact, previous studies have demonstrated efficacy of such models in enhancing student understanding of echocardiographic principles [7, 9].

Given the aforementioned limitations inhibiting widespread adoption of 3D-printed anatomical models in undergraduate medical education, it is important to investigate efficacy of alternative 3D representations of such models. In the present study, the use of 3D-printed anatomical heart models was compared to that of digital 3D Portable Document Format documents (PDFs) accessible on handheld tablets as supplemental learning tools for understanding of echocardiographic principles among preclinical, first-year medical students in the ultrasound portion of a medical anatomy classroom. Students were assigned to utilize one 3D technology, and both model satisfaction and learning quality associated with the technology, as well as understanding of echocardiography, were assessed at various time points. It was hypothesized that both groups would benefit with utilization of their given 3D technology over time, yet the 3D-printed model group would achieve greater model satisfaction, learning quality, and objective echocardiographic knowledge scores relative to the 3D PDF group.

## Materials and Methods

### Institutional Review

This study was deemed exempt by the Case Western Reserve University (CWRU) Institutional Review Board (Submission Number: STUDY20221581).

### Study Design, Setting, and Assignment

**Fig. 1:**
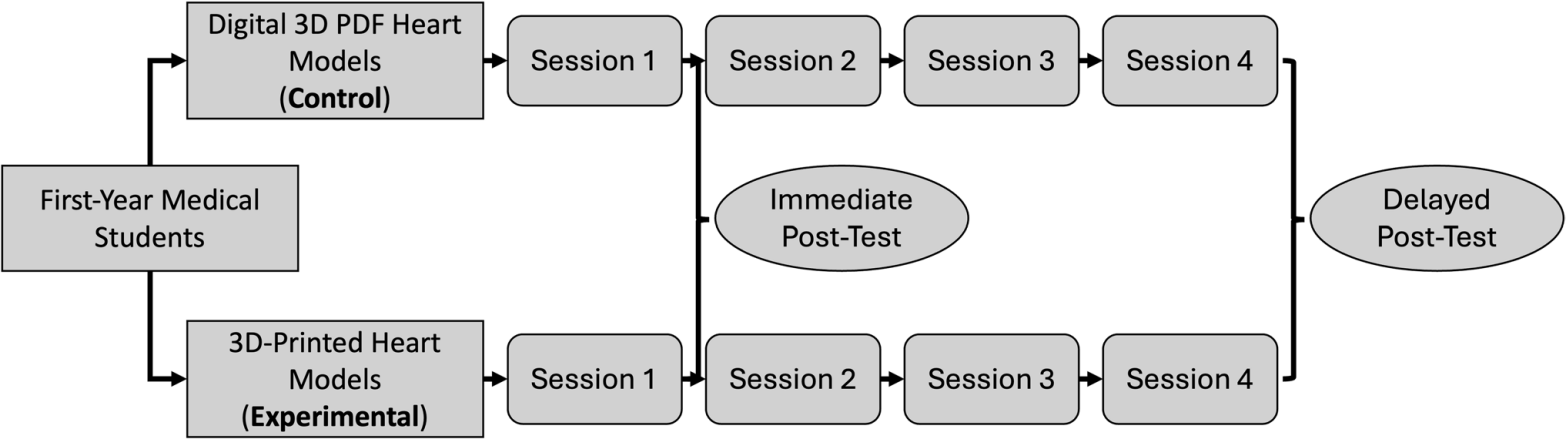
Study design of prospective, single center, unblinded, controlled trial conducted in a living anatomy classroom. Medical students were assigned to utilize either interactive digital 3D PDF heart models (control group) or 3D-printed heart models (experimental group) when performing transthoracic echocardiograms on standardized patients. Students utilized their assigned 3D technology over four thirty-five-minute class sessions. Immediate (after one session) and delayed (after four sessions over two months) post-test assessments were administered to students to assess model satisfaction, learning quality, and echocardiographic knowledge

The present study was designed as a prospective, single center, unblinded, controlled trial with two groups of pre-clinical, first-year medical students assigned to utilize either 3D-printed heart models (experimental) or digital 3D PDFs (control) as learning aids while performing transthoracic echocardiograms (TTEs) on standardized patients (Fig. 1). The investigation took place within the CWRU School of Medicine’s (CWRUSOM) Gross Anatomy, Radiology, and Living Anatomy (GARLA) curriculum [17]. GARLA is the medical school’s longitudinal anatomy and radiology curriculum that takes place during the preclinical phase of student education. GARLA consists of periodic, weekly to biweekly two-hour sessions during which students learn approximately thirty-five minutes of gross anatomy, thirty-five minutes of living anatomy, and thirty-five minutes of radiology pertinent to the preclinical topic students are actively learning during didactic lectures and problem-based learning sessions of CWRUSOM’s Western Reserve 2 (WR2) curriculum [18].

The present study was conducted in the thirty-five-minute living anatomy sub-sessions of the GARLA curriculum, during which students practice ultrasound imaging and physical exam maneuvers on standardized patients. Specifically, the study took place over four thirty-five-minute living anatomy sessions from February to April 2023. The curriculum assigns half of the first-year medical students to Tuesday GARLA sessions, while the other half is assigned to identical Thursday sessions. The Tuesday students were assigned to utilize the digital 3D PDFs accessible on handheld tablets, and the Thursday students were assigned to utilize the 3D-printed heart models, both while performing TTEs on standardized patients. As such, the Tuesday digital 3D PDF group served as the control group, whereas the Thursday 3D-printed heart model group served as the experimental group. Students worked in eight groups of four during any given living anatomy session, with each group having one standardized patient, their assigned 3D technology, and an ultrasound machine with a cardiac probe. Students utilized their assigned 3D technology throughout all four sessions. At the end of the study, students were invited to participate in a voluntary session during which instructors exposed each group to the modality with which the other group had learned.

### 3D Heart Models and PDFs

**Fig. 2:**
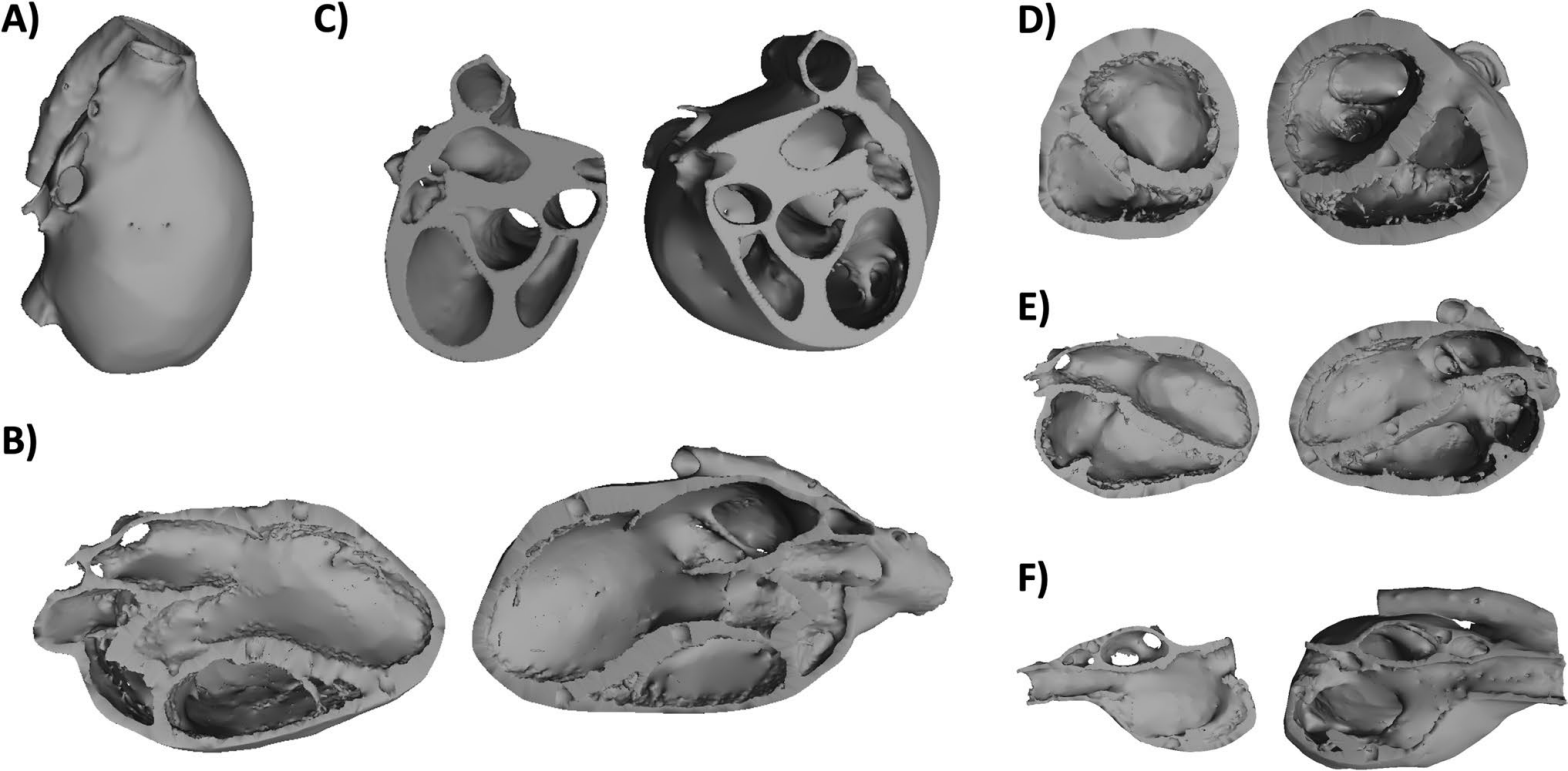
Processed STL files of a healthy human adult heart (**A**) in various echocardiographic cross-sections: **B**) parasternal long axis (PSLA), **C**) parasternal base short axis (PBSA), **D**) parasternal ventricular short axis (PVSA), **E**) apical four-chamber (A4C), and **F**) bicaval (BC)

**Fig. 3:**
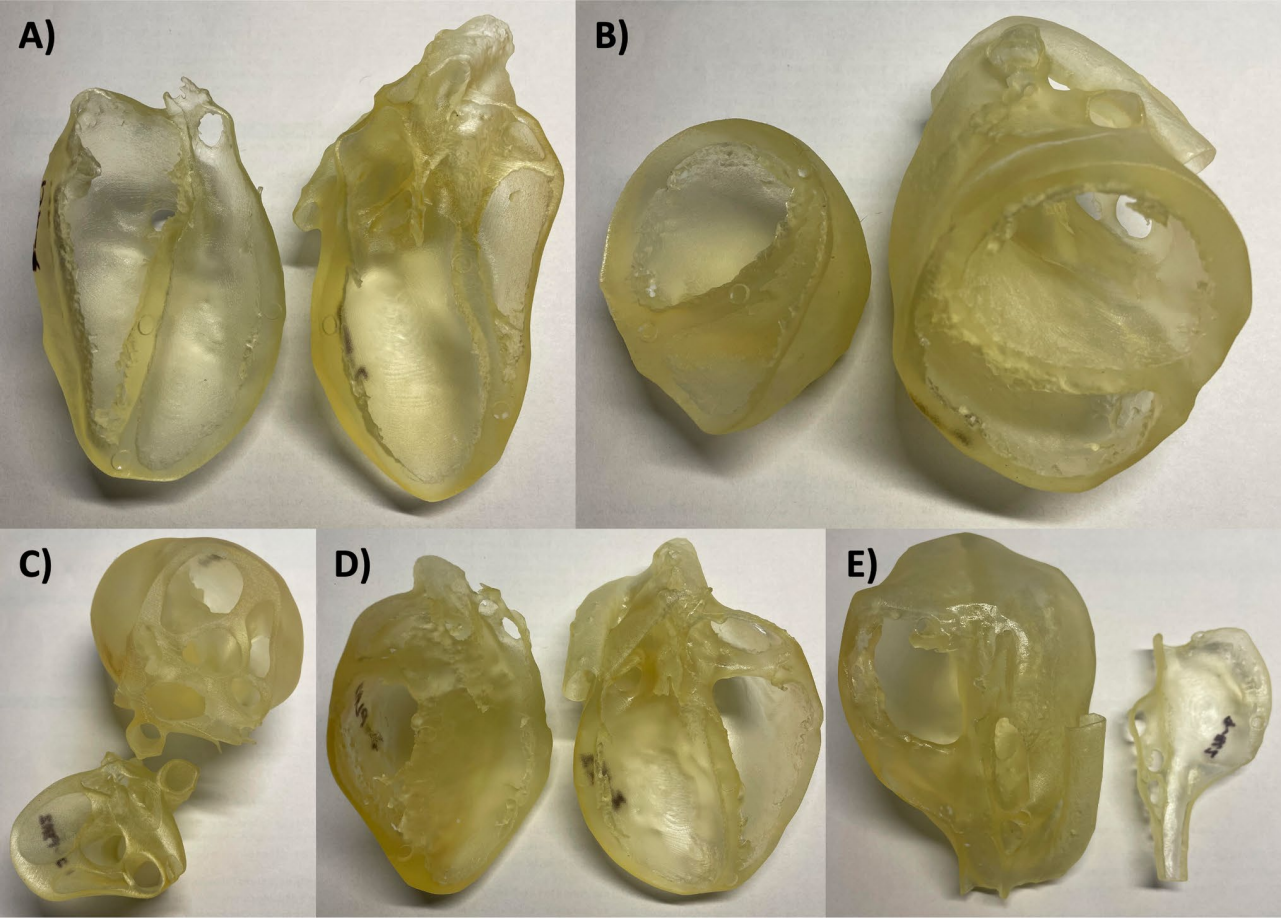
3D-printed heart models in various echocardiographic cross-sections: **A**) parasternal long axis (PSLA), **B**) parasternal base short axis (PBSA), **C**) parasternal ventricular short axis (PVSA), **D**) apical four-chamber (A4C), and **E**) bicaval (BC)

**Fig. 4:**
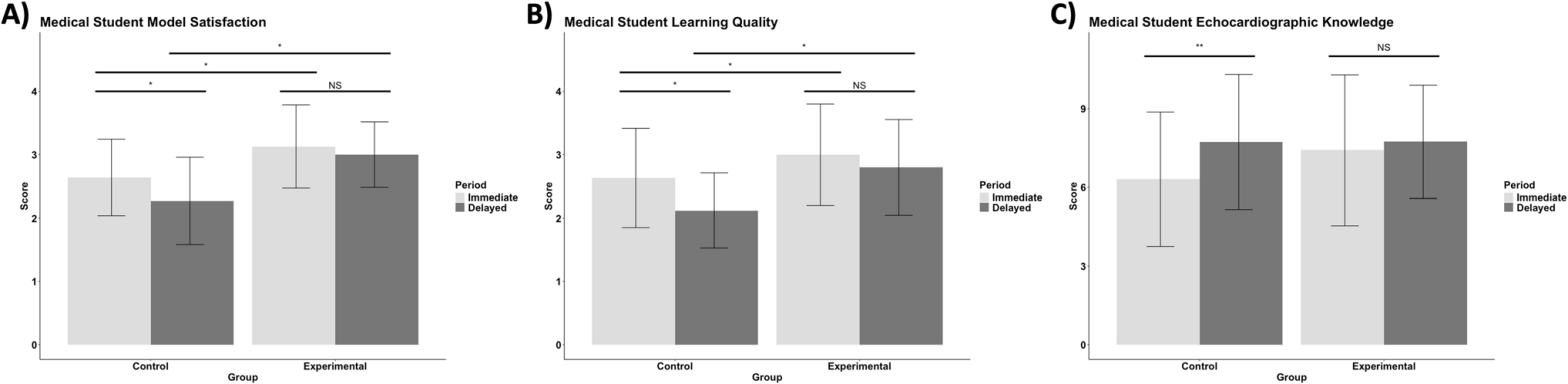
Model satisfaction (**A**), learning quality (**B**), and echocardiographic knowledge (**C**) assessed among medical students utilizing either digital 3D PDF heart models accessible on electronic handheld tablets or 3D-printed models while performing echocardiography on standardized patients in a living anatomy classroom. Light gray shading denotes metrics obtained in the immediate period (after one living anatomy session); dark gray shading denotes metrics obtained in the delayed period (after four living anatomy sessions). Error bars represent standard deviation of the mean score. NS = Not significant. * = Significant at Bonferroni-adjusted p-value threshold of 0.0125. ** = Significant at Bonferroni-adjusted *p*-value threshold of 0.025

Stereolithography (STL) files of a healthy human adult heart (Fig. 2A) were obtained from the open-access Resuscitative TEE (Transesophageal Echocardiography) Project website (ResusMedX LLC) [19] and loaded into Autodesk Meshmixer (Autodesk, Inc., San Francisco, CA, USA) and Mimics (Materialise NV, Leuven, Belgium) for processing. The STL files were analyzed for surface errors before being cross-sectioned into five different projections corresponding to standard echocardiographic views utilized in the clinical setting: 1) parasternal long axis (PSLA; Fig. 2B), 2) parasternal base short axis (PBSA; Fig. 2C), 3) parasternal ventricular short axis (PVSA; Fig. 2D), 4) apical four-chamber (A4C; Fig. 2E), and 5) bicaval (BC; Fig. 2F). The resulting STL files were utilized for both printing of the 3D-printed models (Fig. 3) and creation of digital interactive 3D PDFs in the corresponding cross-sections. For the 3D-printed models, finalized STL files were scaled down to 75% of the original size and fabricated using a Stratasys PolyJet J750 Digital Anatomy Printer (DAP) (Stratsys, Rehovot, Israel). With eight sub-groups of students in each living anatomy session requiring five cross-sections each, a total of forty cross-sections (eighty individual pieces of two pieces per cross-section) were printed, such that each sub-group had an entire set to work with in the classroom. The total internal cost for 3D printing the hearts was $6,240 ($156 per model; $78 per piece), incurred at the Case Western Reserve University Sears think[box] and funded by the Sears think[box] Faculty Fellowship Grant awarded to the investigators in 2022.

The Stratasys J750 DAP was selected for its ability to fabricate anatomical models with precise geometry and desired material properties. The PolyJet technology inherent to the printer operates by jetting and instantly curing tiny droplets of liquid photopolymer to construct models from the provided STL files. The printer can accommodate up to six different materials, which can be used individually or combined to create “Digital Materials” with varying properties. Although several previous studies utilized the cost-effective Fused Deposition Modeling (FDM) technique [20], the thin features of the anatomical heart models and need for impact resistance during handling by learners made the J750 DAP more suitable for this investigation. The 3D-printed heart models were constructed using the semi-rigid polymeric blend of proprietary materials provided by Stratasys. While the J750 DAP has the capacity to print in multi-color, all models for the present study were printed in a monochromatic translucent white color to match the monochromatic nature of the digital interactive 3D PDFs described later. The complexity of the heart models and presence of thin features necessitated the use of support structures during the printing process. These supports were constructed using a soluble material, which was dissolved in a lye solution after 3D printing, leaving only the finalized anatomical models intact.

For the interactive digital 3D PDFs, students were instructed to download the finalized cross-sectional STL files and import these files into the 3D PDF Reader application (Tech Soft 3D) on their handheld electronic tablets. The application allowed students to view and interact with each cross-sectional digital 3D PDF model in real-time while performing echocardiograms. In contrast to the 3D-printed models, the digital 3D PDFs allowed each individual student to have access to all five cross-sectional models at once, while students assigned to the 3D-printed model group shared a set of five anatomical models among the four individuals within each sub-group.

### Evaluation of Model Satisfaction, Learning Quality, and Echocardiographic Knowledge

Medical students in each group were given immediate and delayed post-tests which assessed model satisfaction, learning quality, and echocardiographic knowledge. The post-tests were administered in the living anatomy classroom at the end of the first and fourth living anatomy sessions for the immediate and delayed periods, respectively. Furthermore, the post-tests were completed individually by each student, without any help from other classmates or third-party resources. See Supplements S1 and S2 for electronic copies of the immediate and delayed post-tests, respectively. See Supplements S3 and S4 for the answer keys to the sub-sections of the post-tests assessing echocardiographic knowledge in the immediate and delayed periods, respectively.

Questions 1–2 in each post-test assessed student satisfaction and learning quality with their assigned 3D technology using 4-point Likert scales, respectively. Responses were converted into numerical 1–4 scores, with higher scores representing more positive responses (e.g., “Very dissatisfied” converted to a score of ‘1’; “Excellent” converted to a score of ‘4’), prior to subsequent statistical analysis. Questions 3–5 in each post-test were qualitative free response questions utilized for internal investigation quality control purposes and thus were not subject to subsequent statistical analysis.

Questions 6–14 in both the immediate and delayed post-tests were utilized to assess student understanding of basic anatomical principles and visuospatial orientation underlying echocardiography. As depicted in Supplements S1 and S2, some of the questions assessing echocardiographic knowledge between the post-tests of the immediate and delayed periods were identical, and some of the questions were different. Questions specifically assessed understanding of cardiac anatomy in the parasternal long axis, parasternal short axis, apical four-chamber, and subcostal transthoracic windows. Responses were graded on correctness, and scores were documented from a minimum of zero and maximum of fifteen points for each post-test. Two investigators from the team served as the score recorders to decrease measurement bias, and discrepancies between the two investigators were reconciled prior to subsequent statistical analysis.

The post-test was developed primarily through investigator expertise and prior experience with similar studies; no pilot post-test was administered. Two simplified questions were constructed to assess student satisfaction and learning quality to ensure an acceptable response rate. With regard to echocardiographic knowledge, the investigators revised questions from previous assessments administered in the curriculum to ensure the post-test of the present study focused on basic anatomical principles and visuospatial orientation.

### Statistical Analysis

Cronbach’s alpha coefficient and Kendall’s tau-b were calculated to evaluate reliability of the post-test questionnaire administered to students. Numeric scores for model satisfaction, learning quality, and echocardiographic knowledge were compared using two-way analysis of variance (ANOVA) with replication tests, followed by post-hoc independent samples t-tests with Bonferroni correction for multiple comparisons when indicated. Scores were compared within each group from the immediate to the delayed periods to assess for improvement in model satisfaction, learning quality, and objective understanding of echocardiographic principles over the study period. Furthermore, the scores were compared between groups at both the immediate and delayed periods to assess for superiority of the experimental 3D-printed heart models relative to the control digital 3D PDFs with respect to the same endpoints. Statistical analysis was conducted in Microsoft Excel (Version 16.90), and illustrations were created using the R programming language (Version 4.4.1) and the RStudio Integrated Development Environment (Version 2024.04.2 + 764). Statistical significance of the two-way ANOVA with replication tests was determined at a p-value threshold of 0.05, with Bonferroni-adjusted p-value thresholds calculated post-hoc given the variable number of comparison tests.

## Results

### Study Sample, Response Rate, and Post-Test Reliability

One-hundred fifty medical students were surveyed in the study, and seventy-five medical students each were assigned to utilize either the 3D-printed heart models (experimental group) or the digital 3D PDF heart models (control group).

In the immediate period, there were 46 (61.3%) scored responses for the question assessing model satisfaction in the control group and 70 (93.3%) scored responses for this question in the experimental group. Also in the immediate period, there were 49 (65.3%) scored responses for the question assessing learning quality in the control group and 73 (97.3%) scored responses for this question in the experimental group. In the delayed period, there were 74 (98.7%) scored responses for the question assessing model satisfaction in the control group and 68 (90.7%) scored responses in the experimental group. For the question assessing learning quality in the delayed period, there were 75 (100.0%) scored responses in the control group and 65 (86.7%) scored responses in the experimental group.

With regard to the questions of the post-tests assessing echocardiographic knowledge in the immediate period, there were 50 (66.6%) scored responses in the control group and 58 (77.3%) scored responses in the experimental group. In the delayed period, there were 73 (97.3%) scored responses in the control group and 68 (90.7%) scored responses in the experimental group. Cronbach’s alpha coefficient was calculated to be 0.74 for the post-test, suggesting acceptable reliability. From Kendall’s tau-b analysis, model satisfaction and learning quality scores had a strong, positive correlation among all students (τ_b_ = 0.636; *p* < 0.001). No correlation was found between model satisfaction and echocardiographic knowledge (τ_b_ = 0.064; *p* = 0.225) or between learning quality and echocardiographic knowledge (τ_b_ = 0.043; *p* = 0.409).

### Model Satisfaction and Learning Quality

Two-way ANOVA with replication tests for mean model satisfaction and learning quality scores demonstrated statistically significant differences among groups with respect to both the 3D technology assigned (*p* < 0.001 and *p* < 0.001, respectively) and period of the study (*p* = 0.002 and *p* < 0.001, respectively). The interaction effect between the 3D technology assigned and period of the study was not found to be statistically significant for either model satisfaction (*p* = 0.126) or learning quality (*p* = 0.089). Nevertheless, post-hoc testing was pursued for each endpoint, given the significant main factorial effects demonstrated by two-way ANOVA testing. The Bonferroni-adjusted p-value threshold was calculated to be 0.0125, given that four post-hoc comparison tests were conducted.

From post-hoc analysis, students in the experimental group were found to report greater mean model satisfaction scores relative to the control group in the immediate period (3.13 ± 0.66 vs. 2.64 ± 0.60, respectively; *p* < 0.001; Fig. 4A). Students in the experimental group also reported greater mean model satisfaction scores relative to the control group in the delayed period (3.00 ± 0.52 vs. 2.27 ± 0.69, respectively; *p* < 0.001; Fig. 4A). Students in the control group reported lower mean satisfaction scores from the immediate period to the delayed period (2.64 ± 0.60 vs. 2.27 ± 0.69, respectively; *p* = 0.001; Fig. 4A), whereas mean model satisfaction scores of the experimental group were similar between the immediate and delayed periods (3.13 ± 0.66 vs. 3.00 ± 0.52, respectively; *p* = 0.102; Fig. 4A).

Regarding learning quality, post-hoc analysis found that students in the experimental group reported greater mean scores relative to the control group in the immediate period (3.00 ± 0.80 vs. 2.63 ± 0.78, respectively; *p* = 0.007; Fig. 4B). Students in the experimental group also reported greater mean learning quality scores relative to the control group in the delayed period (2.80 ± 0.75 vs. 2.12 ± 0.59, respectively; *p* < 0.001; Fig. 4B). From the immediate period to the delayed period, mean learning quality scores of the control group decreased (2.63 ± 0.78 vs. 2.12 ± 0.59, respectively; *p* < 0.001; Fig. 4B). In contrast, mean learning quality scores of the experimental group were similar between the immediate and delayed periods (3.00 ± 0.80 vs. 2.80 ± 0.75, respectively; *p* = 0.067; Fig. 4B).

### Objective Echocardiographic Understanding

A two-way ANOVA with replication test for mean echocardiographic knowledge score demonstrated a statistically significant difference among groups with regard to only the period of the study (*p* = 0.008). However, the two-way ANOVA test did not find a statistically significant difference in mean echocardiographic knowledge among groups with regard to the 3D technology assigned (*p* = 0.091) or the interaction effect between the 3D technology assigned and period of the study (*p* = 0.092). Post-hoc testing for mean echocardiographic knowledge was thus conducted with respect to only the period of the study, and the Bonferroni-adjusted p-value threshold was calculated to be 0.025, given that two post-hoc comparison tests were conducted.

From the immediate to the delayed period, post-hoc analysis found that mean echocardiographic knowledge scores increased in the control group (6.31 ± 2.56 vs 7.73 ± 2.57, respectively; *p* = 0.002; Fig. 4C). In the experimental group, mean echocardiographic knowledge scores were similar between the immediate and delayed periods (7.41 ± 2.88 vs. 7.74 ± 2.16, respectively; *p* = 0.238; Fig. 4C).

## Discussion

The present study was a prospective, single center, unblinded, controlled trial comparing the longitudinal use of either 3D-printed heart models or interactive digital 3D PDF heart models among medical students when learning echocardiographic principles in a living anatomy classroom with standardized patients. To our knowledge, this is the first medical education investigation directly comparing use of 3D-printed models to interactive digital 3D PDFs as learning aids. The key findings of the study are two-fold: 1) long-term utilization of interactive digital 3D PDF heart models accessible on handheld tablets is non-inferior to 3D-printed heart models with respect to objective learner understanding of basic anatomical principles and visuospatial orientation underlying echocardiography, and 2) 3D-printed heart models may provide medical students with greater satisfaction and learning quality relative to interactive digital 3D PDF heart models. These findings not only support growing evidence behind the integration of 3D technologies into the medical education environment for learning of anatomical and clinical imaging principles, but also suggest that digital 3D PDF models accessible on handheld tablets may be viable alternatives to 3D-printed anatomical models.

Previous studies have demonstrated efficacy of 3D-printed heart models in the medical education setting for learning echocardiography [7, 9]. In 2019, an investigation conducted by Ochoa et al. [9] compared image acquisition and knowledge retention of echocardiography among twenty medical students assigned to utilize either 3D-printed heart models or traditional didactic lecture methods. Findings of the Ochoa et al. study suggested similar skill acquisition and knowledge retention observed between both groups, and the investigators concluded that 3D-printed heart models may be more effective when accounting for the greater costs and decreased accessibility of traditional lecturers. More recently, a study conducted by Salewski et al. [7] in 2022 demonstrated increased visuospatial understanding of basic echocardiographic principles among seventy-seven medical students utilizing 3D-printed heart models compared to seventy-six students utilizing conventional anatomical teaching methods. Since the present study did not have a comparison group solely utilizing traditional teaching methods, it is difficult to discern whether the present study’s echocardiographic knowledge post-assessment findings align with those of the prior investigations [7, 9]. However, in the present study, mean model satisfaction and learning quality scores in the experimental group of medical students utilizing 3D-printed heart models were generally on the positive end of the spectrum in both the immediate and delayed periods. The present study therefore provides positive student opinion support to the findings supporting learning efficacy with 3D-printed heart models of the previous investigations.

In contrast to 3D-printed heart models, previous studies investigating utilization of interactive digital 3D PDF models as learning aids in echocardiography are scarce, if not nonexistent, in the literature. Previous investigations have instead compared use of digital 3D PDF models to conventional teaching methods in other anatomical applications [21, 22]. In 2020, a study conducted by Chekrouni et al. [21] suggested improved understanding of embryology in biomedical students utilizing 3D PDF models of human embryological development. Similarly, an investigation conducted by Eroğlu et al. [22] in 2023 found superior objective test performance in medical students utilizing interactive 3D PDF models relative to those utilizing 2D atlases when learning liver and male genitalia anatomy. Although as previously mentioned, the present study did not possess a conventional teaching method comparison group, findings did suggest similar efficacy between digital 3D PDF models and 3D-printed models in learning echocardiographic principles. This aligns with findings of the studies conducted by Chekrouni et al. [21] and Eroğlu et al. [22]. It is important to note, however, that the model satisfaction and learning quality ratings of the digital 3D PDF model group in the present study were on the negative end of the spectrum in both the immediate and delayed periods, and the ratings in fact decreased from the immediate to the delayed period. Therefore, although objective understanding may be effectively achieved with interactive digital 3D PDF models in learning echocardiography, the low model satisfaction and learning quality ratings suggest that students may not end up using such models if they are integrated into the medical education environment.

The similar mean echocardiographic knowledge score of the control and experimental groups in the present study’s delayed period support efficacy of 3D technologies in medical education for learning supplements in understanding echocardiography. A previous investigation analyzing learning of echocardiography among students pointed to the need to reconcile 2D image projections with cardiac anatomy as the primary difficulty of grasping relevant echocardiographic concepts [15]. However, the previous study also reported that access to pedagogical equipment such as an “anatomical dummy of the heart” aided students in performing this reconciliation. This is likely the reason behind the present study demonstrating similar efficacy of both 3D technologies in learning echocardiography, as students may manipulate models in real-time while performing echocardiograms to reconcile the distinct 2D cross-sections with the 3D models. This real-time reconciliation may increase visuospatial understanding of cardiac anatomy in response to dynamic changes of ultrasound probe placement, angulation, and translation. Such reconciliation readily aligns itself with constructivist learning theory, in which it is viewed that learners build new ideas based upon current or past knowledge through interaction with meaning activities (i.e., 3D technologies) [23].

The decrease in model satisfaction and learning quality scores in the interactive digital 3D PDF model group from the immediate to the delayed period, as well as the lower scores of this group in these endpoints when compared to that of the 3D-printed model group in both periods, is an area of concern. There are several reasons that may explain these discrepancies. Firstly, the learning curve for properly utilizing interactive digital 3D PDF models may be steeper relative to the 3D-printed models, as the PDFs require students to use tools within the software program to manipulate models on a handheld electronic tablet application. The steeper learning curve may also explain the improvement in echocardiographic knowledge from the immediate to the delayed period in the control group, as students may have become more familiar with the handheld tablet application over subsequent sessions or outside of the classroom (whereas accessibility of the 3D-printed models was limited outside of the classroom). In contrast, the 3D-printed models may be easier to manipulate, as they fit within the students’ hands and can be readily rotated and translated to conform to the given 2D cross-section appearing on the ultrasound image. Similarly, the 3D PDF models on handheld tablets may have been more burdensome to navigate with a student’s free hand if their other hand was occupied by the cardiac probe to simultaneously perform the echocardiogram. With the 3D-printed models, on the other hand, students can easily manipulate a 3D cross-section with their free hand while simultaneously manipulating the ultrasound probe with their other hand.

These differences in handling inherently associated with use of 3D-printed heart models compared to that of 3D digital PDFs may also highlight the importance of tactile interaction with 3D technological aids when learning anatomy. In the present study, fine, thin model features present in the STL files were difficult to reproduce during the printing process, due to the semi-rigid polymer being utilized and these features subsequently collapsing during printing. As a result, the 3D-printed heart models likely had suboptimal spatial resolution when compared to that of the interactive 3D digital PDFs, which were created by simply importing the STL files into the tablet application without such loss of fine detail. Thus, the greater model satisfaction and learning quality scores of the experimental group relative to the control may suggest that tactile interaction with a 3D technology may be more important than spatial resolution. Similarly, the differences in scores may suggest that the burdensome task of using two hands to navigate the tablet’s electronic application may undermine the enhanced spatial resolution of the interactive digital 3D PDF models.

### Strengths, Limitations, and Areas of Future Investigation

Strengths of the present study included the novel design comparing two alternative modalities of 3D technologies, large sample size of one-hundred fifty medical students, and longitudinal assessment of model satisfaction, learning quality, and echocardiographic knowledge. Furthermore, the response rates of the post-tests reflected the majority of medical students assigned to both groups in the immediate and delayed periods of the study. However, this study is not without limitations. As previously mentioned regarding study design, there was no comparison group in which medical students solely utilized conventional anatomical teaching methods (e.g., didactic lectures and 2D atlases) without any 3D technology. The investigators decided to forego such a comparison group to prioritize equity. The lack of a comparison group rendered it impossible to discern whether either 3D technology utilized had superior model satisfaction, learning quality, and echocardiographic knowledge scores compared to traditional teaching alone. Similarly, a cross-over paradigm may have been beneficial for students to directly compare both 3D technologies and more clearly establish a favored model, yet this was not performed due to curricular time constraints. The present study also did not provide a pre-test prior to the living anatomy course, and there was thus no baseline knowledge of echocardiography established for comparison to the immediate and delayed post-test assessments. The questions that were non-identical between the immediate and delayed post-tests were implemented to mitigate the effects of omitting such a pre-test on the internal validity of this study. The study also did not include a post-test administered after the four-week study period, which may have been beneficial for establishing long-term knowledge retention using each 3D technology. Additional limitations of the present study involved the aforementioned unfamiliarity of students with the 3D PDF Reader application on their handheld tablets (the likely frustrating initial experience of which may explain low response rates of the post-test in the immediate period for the control group), small effect sizes, warping of the 3D-printed models during production resulting in suboptimal model quality, and the lack of pairing in statistical analysis to track scores of the same individuals over time from the immediate to the delayed period. Areas of future investigation should address these limitations, in addition to incorporating additional modalities of 3D technology (e.g., virtual reality or augmented reality) and historical controls for comparison, assessing alternative anatomical or clinical applications with 3D PDF models (e.g., hepatobiliary, pulmonary, or renal systems), highlighting important anatomical features on 3D models using color shades or labels, performing a cost–benefit analysis to assess feasibility of implementing 3D models in the classroom, and comparing 3D interventions as learning aids in identifying and understanding pathophysiology observed on echocardiography.

## Conclusions

In a prospective, single center, unblinded, controlled trial conducted in a living anatomy classroom, utilization of interactive digital 3D PDF heart models accessible on electronic handheld tablets achieved similar echocardiographic knowledge scores relative to 3D-printed heart models in understanding of echocardiographic principles among medical students. However, there were decreased model satisfaction and learning quality scores with medical student utilization of the 3D PDF heart models relative to that of the 3D-printed heart models. Although 3D PDF heart models may have similar efficacy to 3D-printed heart models in supplementing medical student learning of echocardiography, students may prefer 3D-printed models over 3D PDFs. Future studies should continue investigating alternative 3D technological modalities in supplementing medical student learning of anatomy and ultrasound imaging.

## Supplementary Information

Below is the link to the electronic supplementary material.

Supplementary file1 (PDF 963 KB)

Supplementary file2 (PDF 1318 KB)

Supplementary file3 (PDF 1404 KB)

Supplementary file4 (PDF 1310 KB)

## Data Availability

The participants of this study did not give written consent for their data to be shared publicly, so due to the sensitive nature of the research supporting data is not available.
