# Supplementary material for: Comparing the Effectiveness of Digital 3D PDF vs. 3D-Printed Heart Models as Learning Aids for Echocardiography in Medical Students
Source: Med Sci Educ. 2025 Apr 29;35(4):1983–92. doi: 10.1007/s40670-025-02392-x (PMC12532530; doi:10.1007/s40670-025-02392-x)

DELAYED POST-TEST

1. Rate your level of satisfaction with using the 3D printed model/interactive PDF to learn echocardiographic structures. (Please circle one.)

   Very dissatisfied   Dissatisfied   Satisfied   Very satisfied

2. Rate the quality of the 3D printed model/interactive PDF as a tool for learning echocardiographic structures.

   Poor   Fair   Good   Excellent

3. Explain why you rated the quality of the 3D printed model/interactive PDF as you did.

4. Describe the benefits of using the 3D printed model/interactive PDF to learn echocardiography.

5. Describe how your learning could be improved using the 3D printed model/interactive

THE FOLLOWING QUESTIONS ASSUME A CARDIAC ORIENTATION WITH TRANSDUCER INDICATOR DIRECTOR TOWARD THE PATIENT'S LEFT FOR SHORT-AXIS WINDOWS AND TOWARD THE PATIENT'S HEAD FOR LONG-AXIS WINDOWS

6) Identify the following structures (see image).

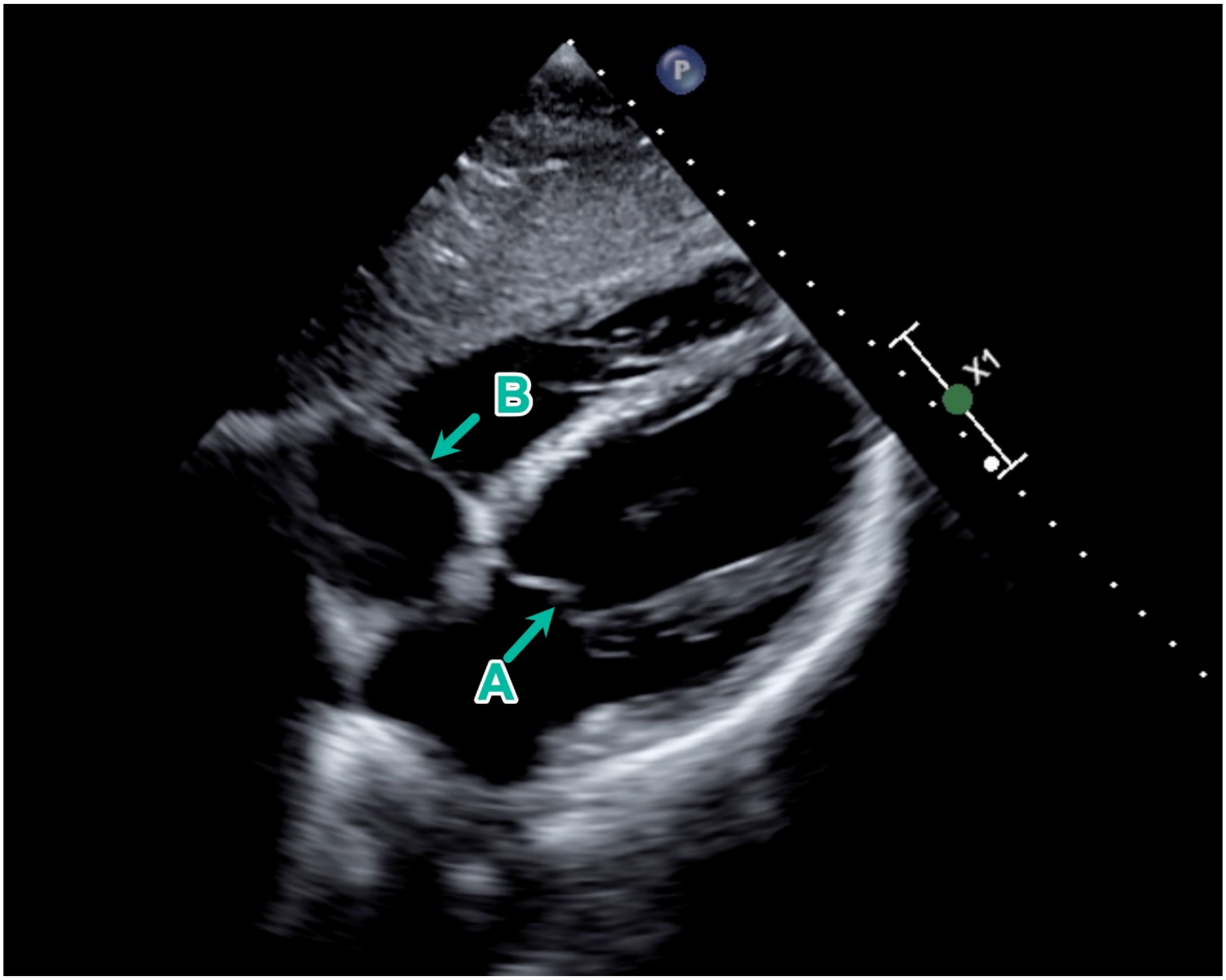

7) In order to visualize the aortic outflow tract, what transducer movement would be required from the current window (see image)?

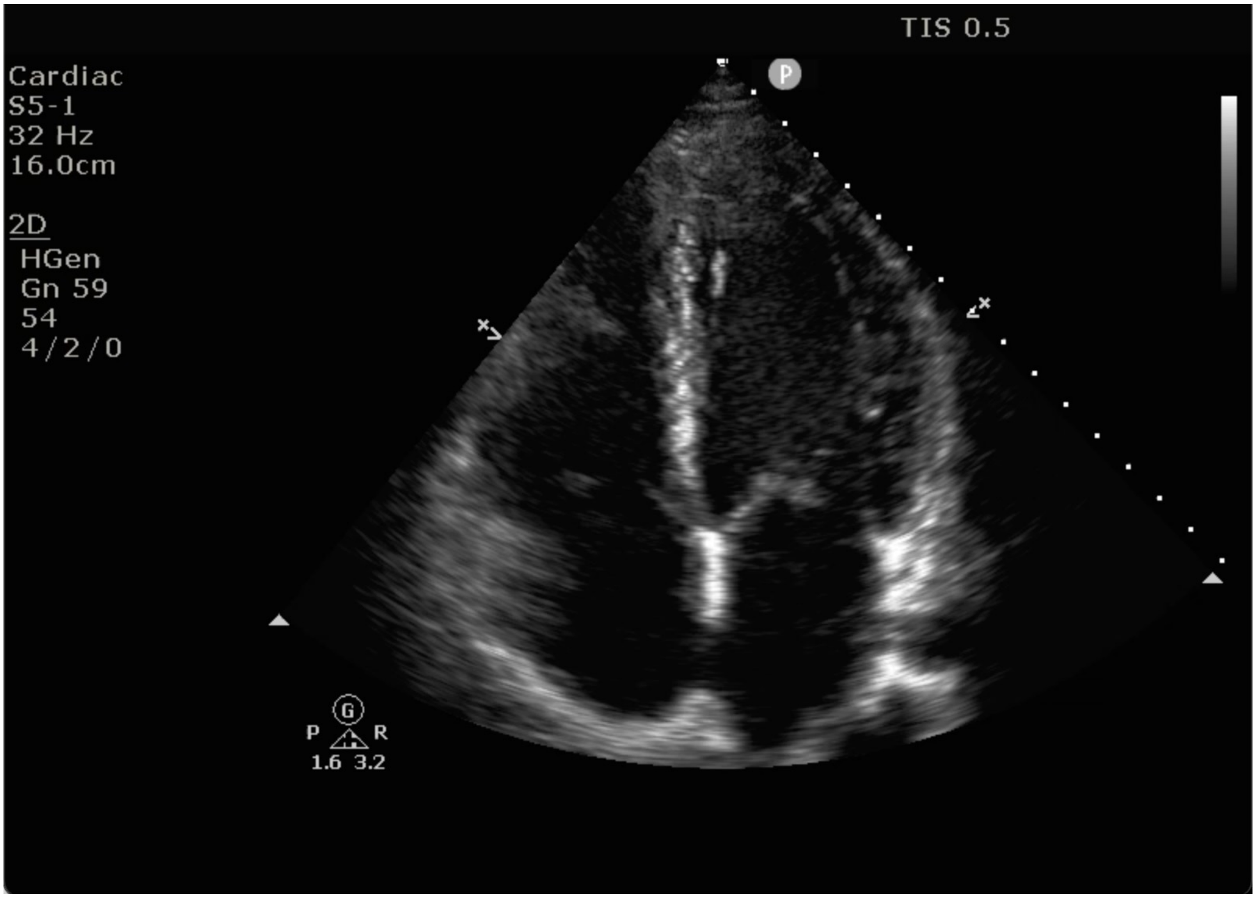

8) Identify the following structures (see image).

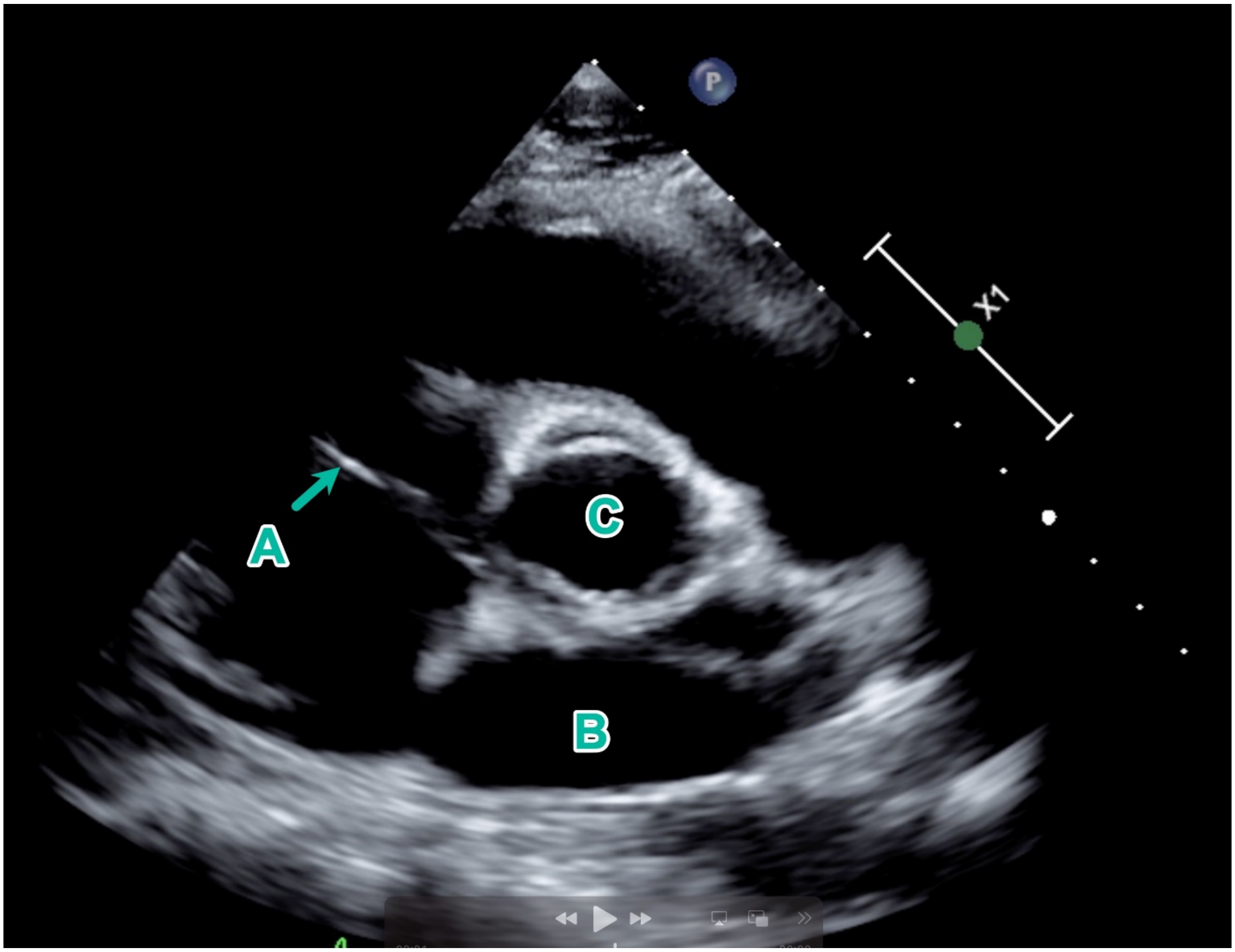

9) What axis of the heart is shown in the image?

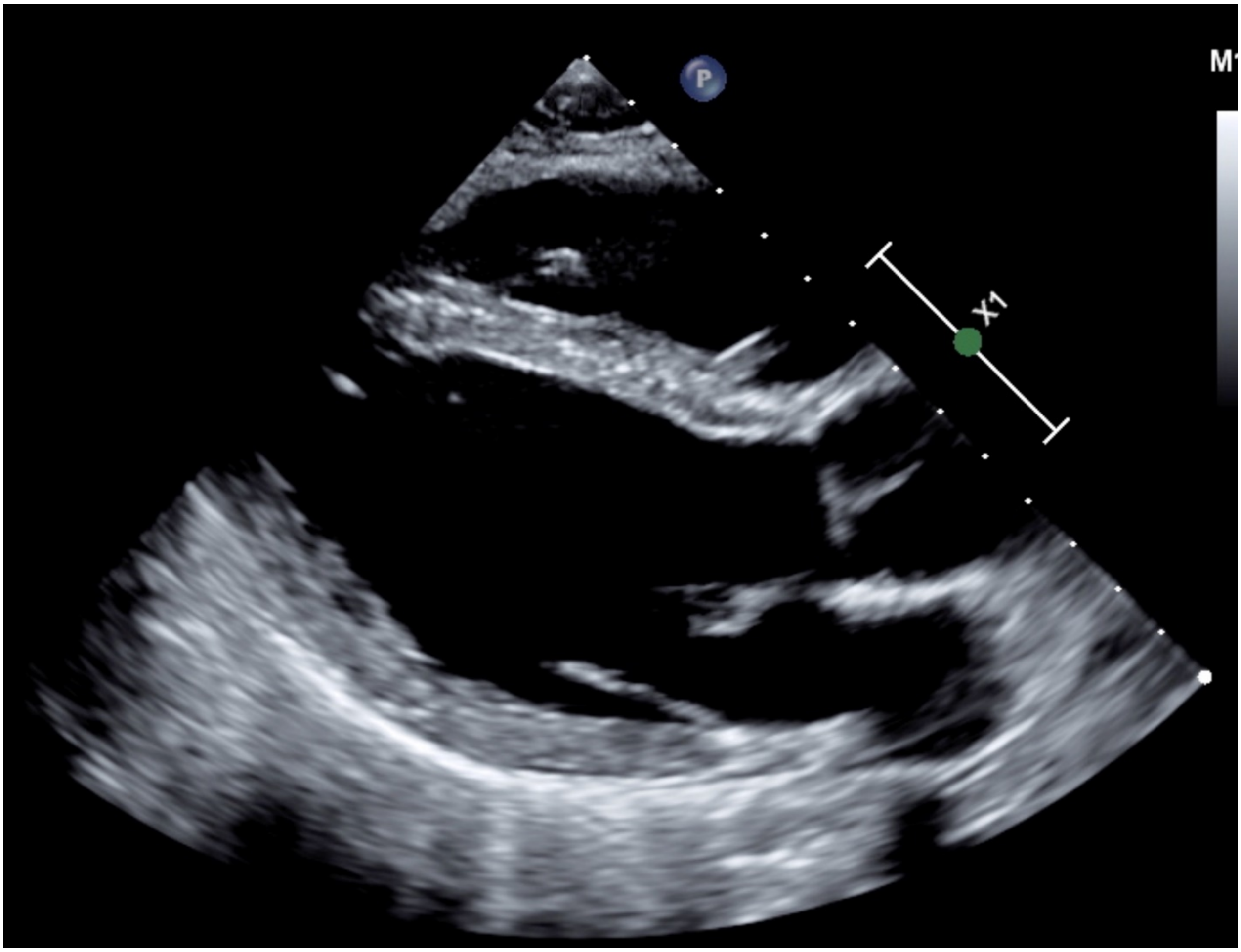

10) Identify the following structures (see image).

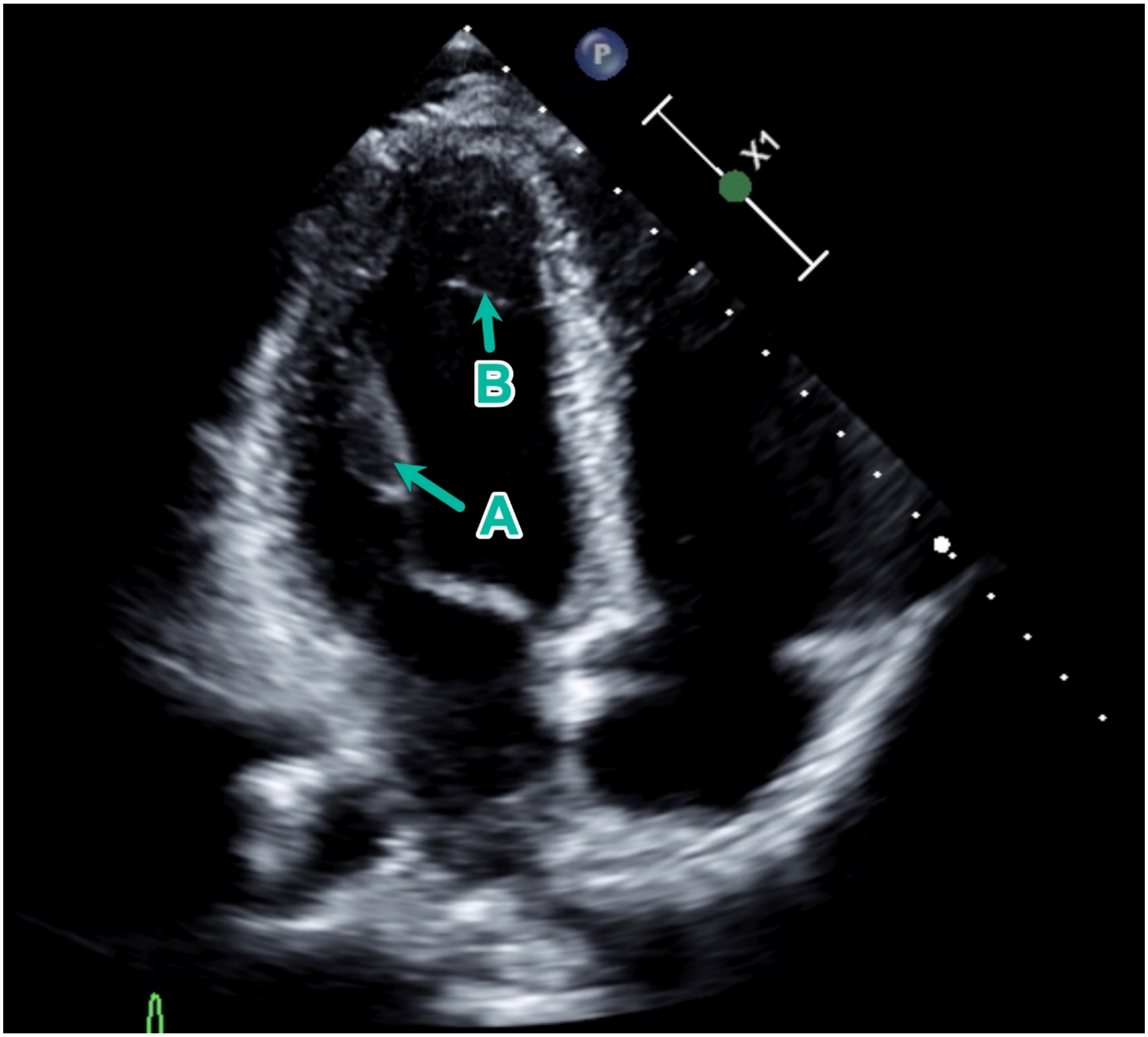

11) In order to visualize the aortic valve in short axis, what transducer movement would be required from the current window (see image)?

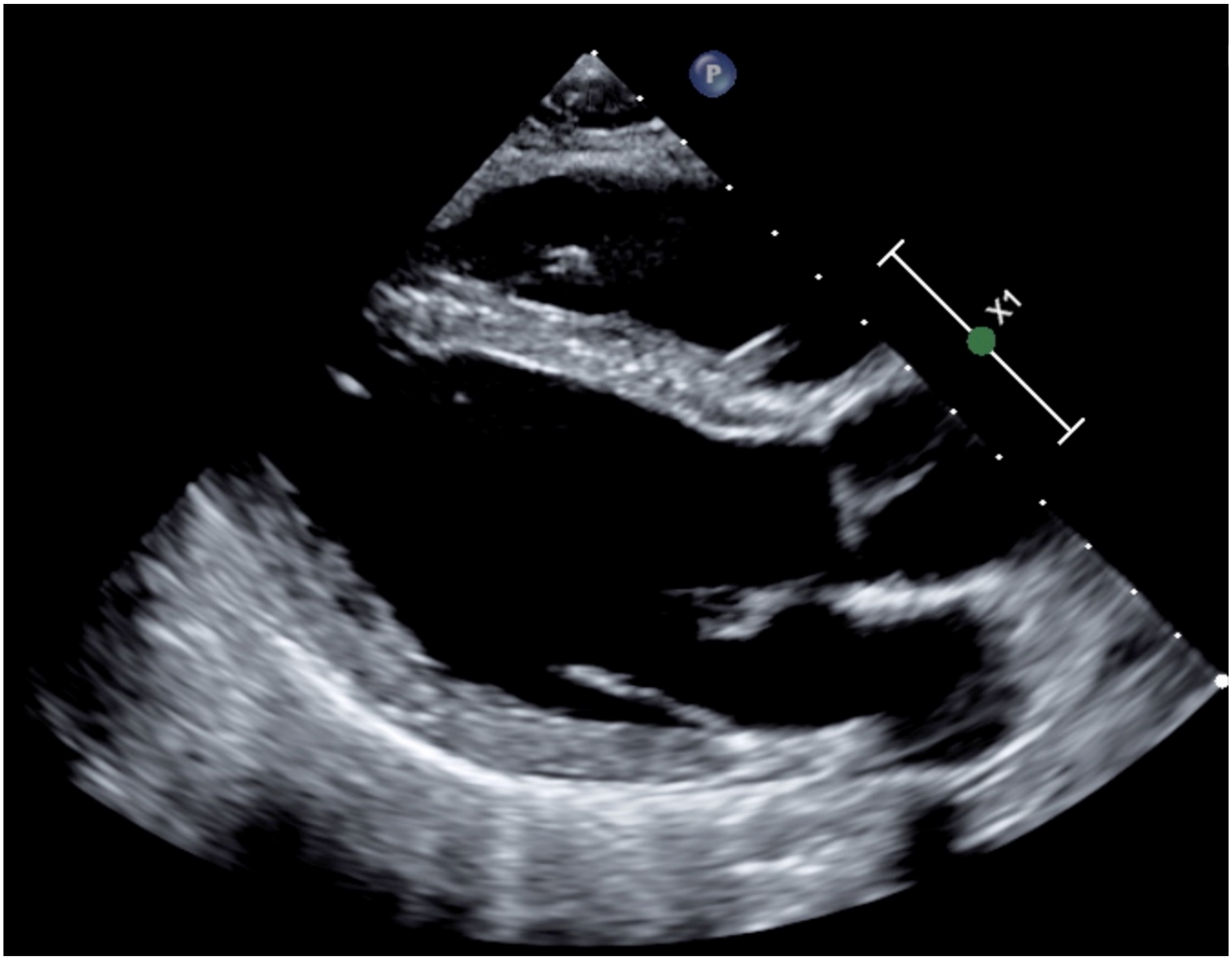

12) Identify the following structures (see image).

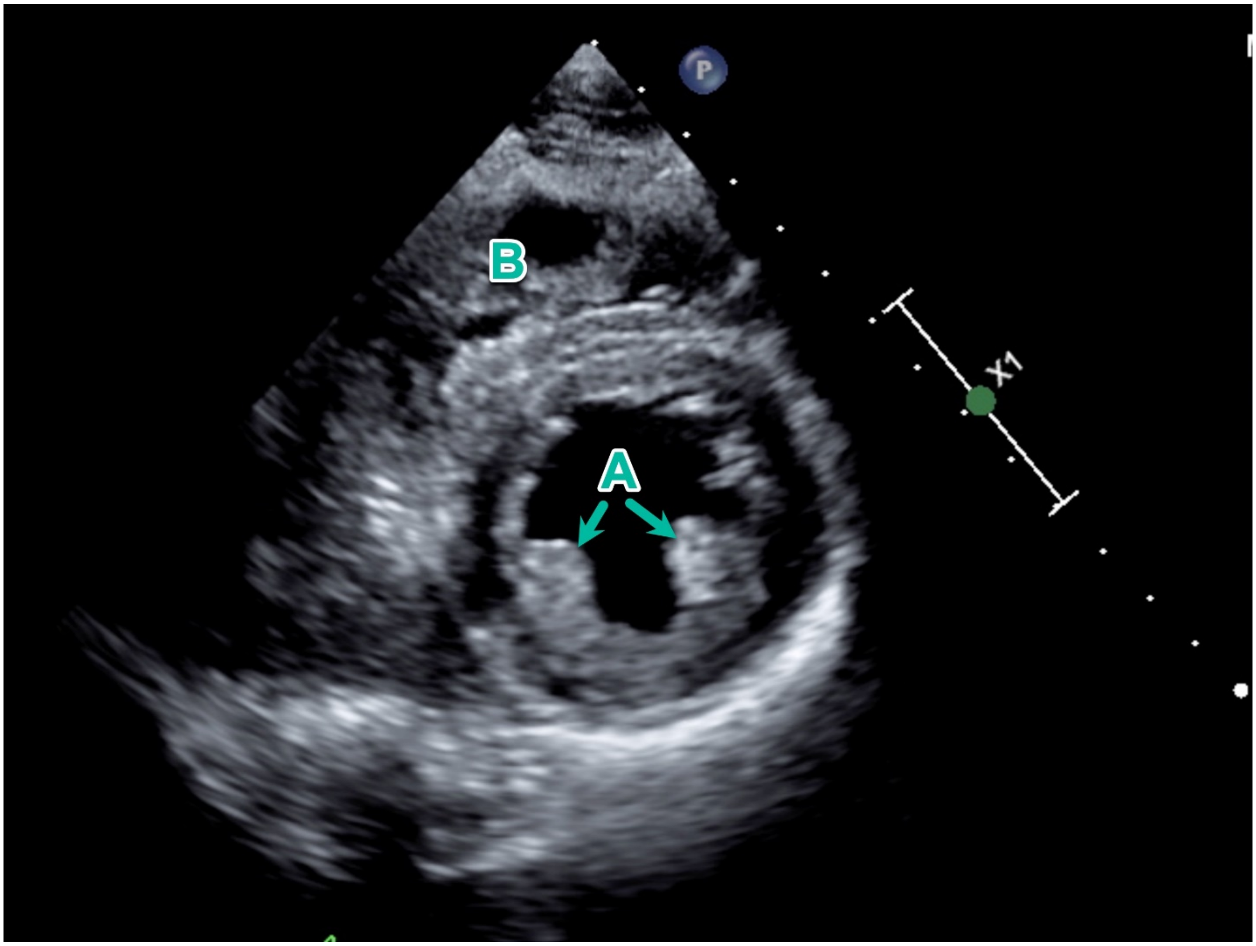

13) In order to obtain a window showing LV inflow and outflow, what transducer movement would be required from the current window?

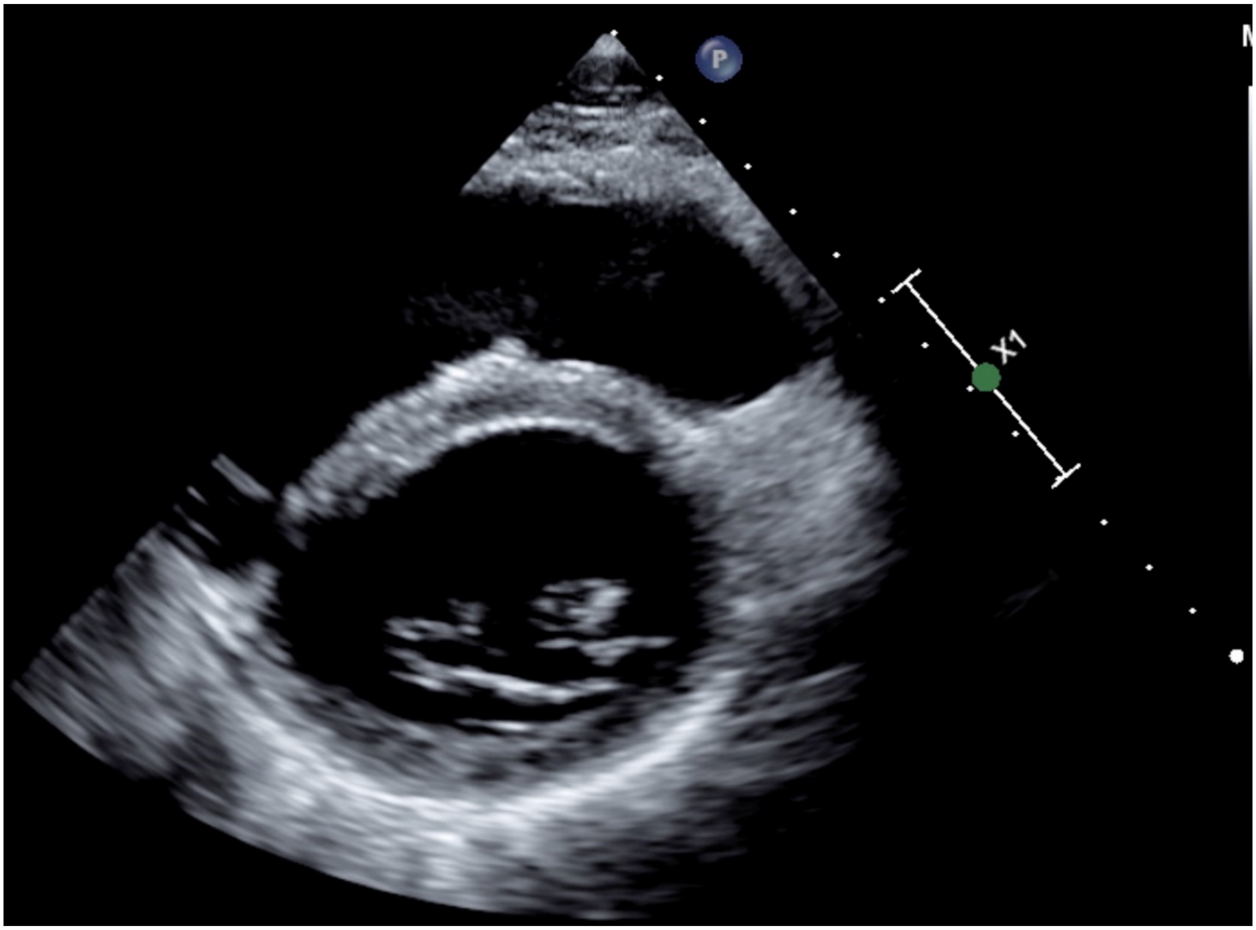

14) This image was obtained by placing the transducer in what anatomic location?

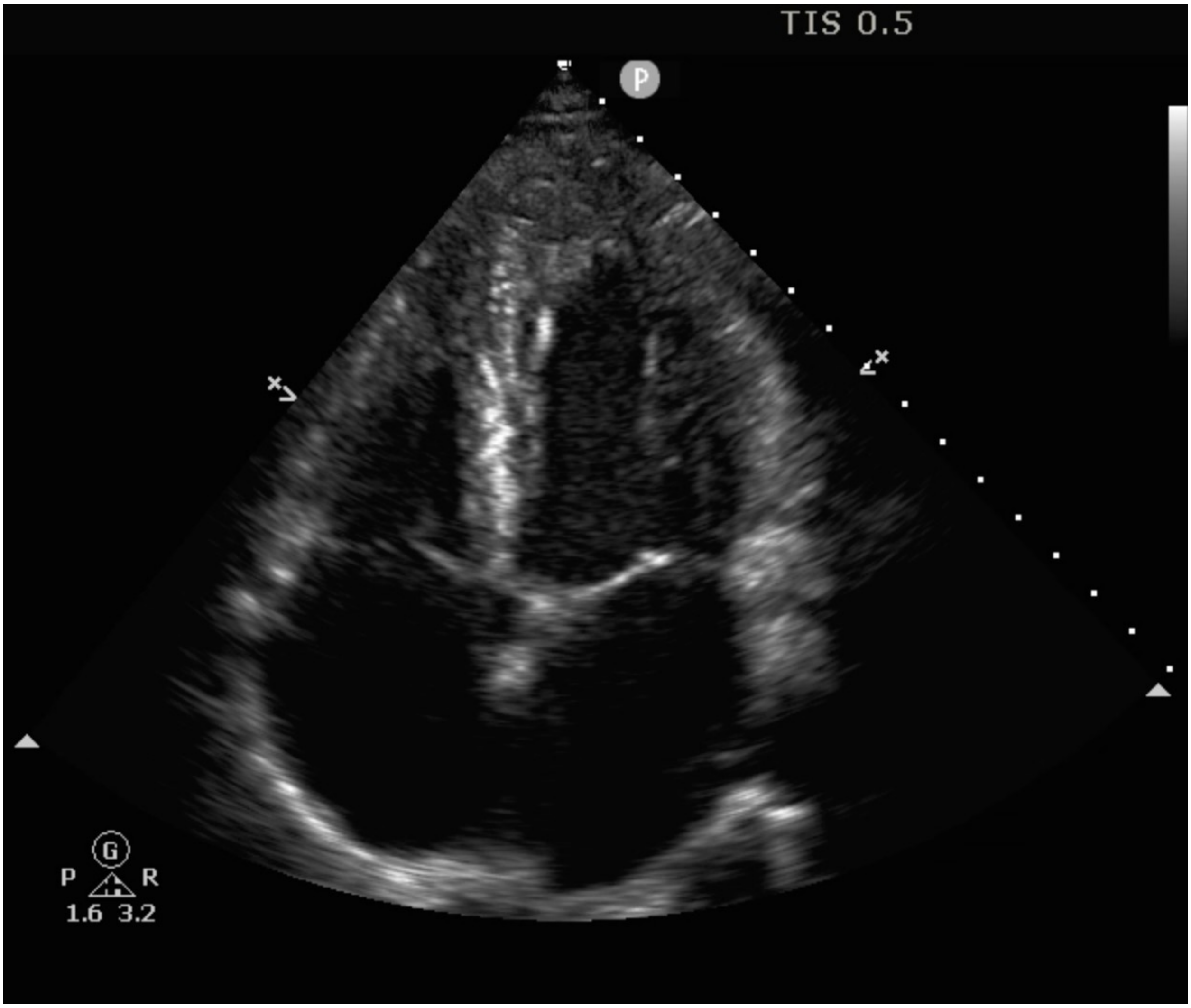

Supplement: Supplementary file 2 — Supplementary file2 (PDF 1318 KB) [file 40670_2025_2392_MOESM2_ESM.pdf]
