# Supplementary material for: Comparing the Effectiveness of Digital 3D PDF vs. 3D-Printed Heart Models as Learning Aids for Echocardiography in Medical Students
Source: Med Sci Educ. 2025 Apr 29;35(4):1983–92. doi: 10.1007/s40670-025-02392-x (PMC12532530; doi:10.1007/s40670-025-02392-x)

IMMEDIATE POST-TEST KEY

6) In order to visualize the apex of the heart, what transducer movement would be required from the current window (see image)? Answer: Caudal.

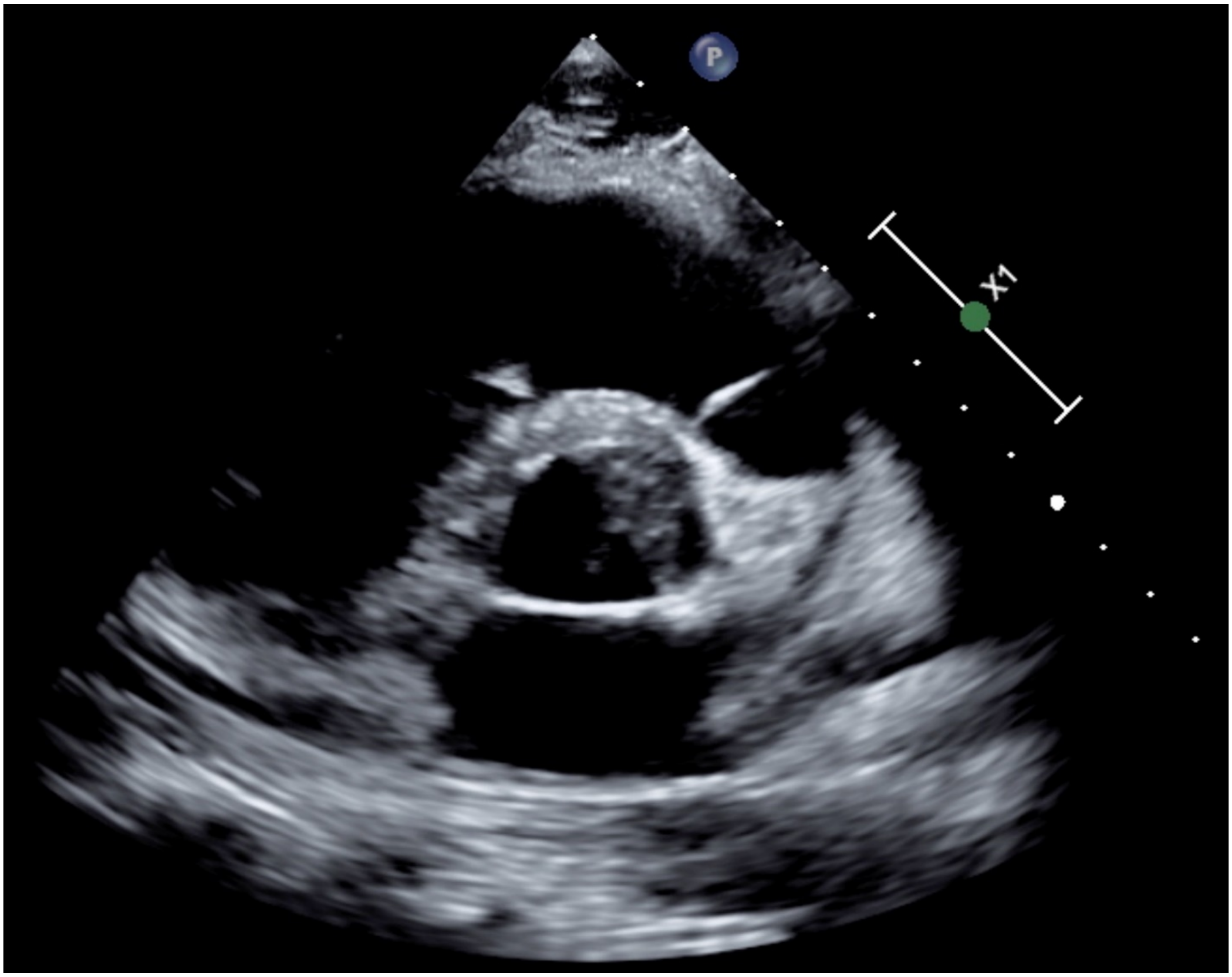

7) Identify the following structures (see image). Answer: A. Right Ventricle. B. Left Ventricle. C. Aortic outflow tract.

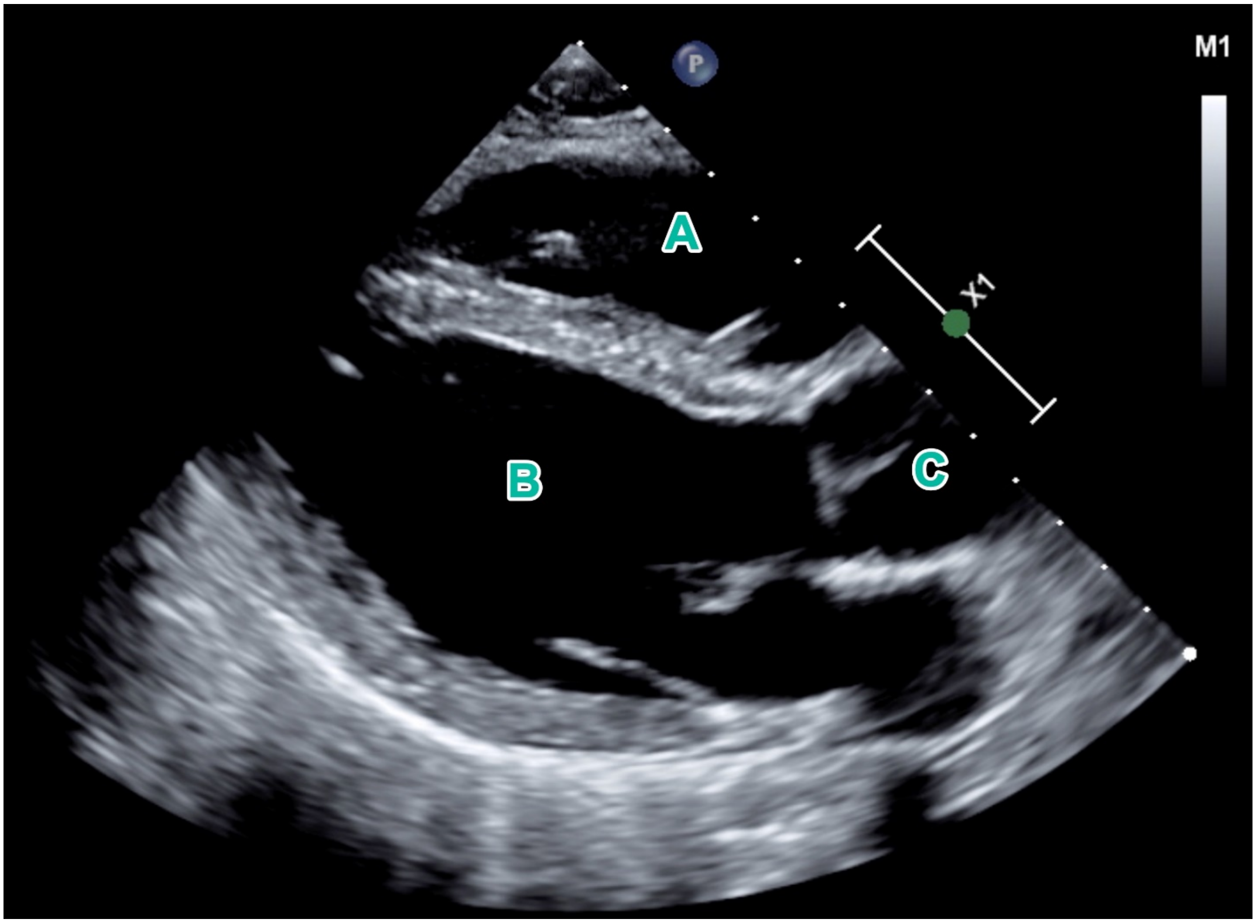

8) Identify the following structures (see image). Answer: A. Right Atrium. B. Left Ventricle. C. Mitral Valve.

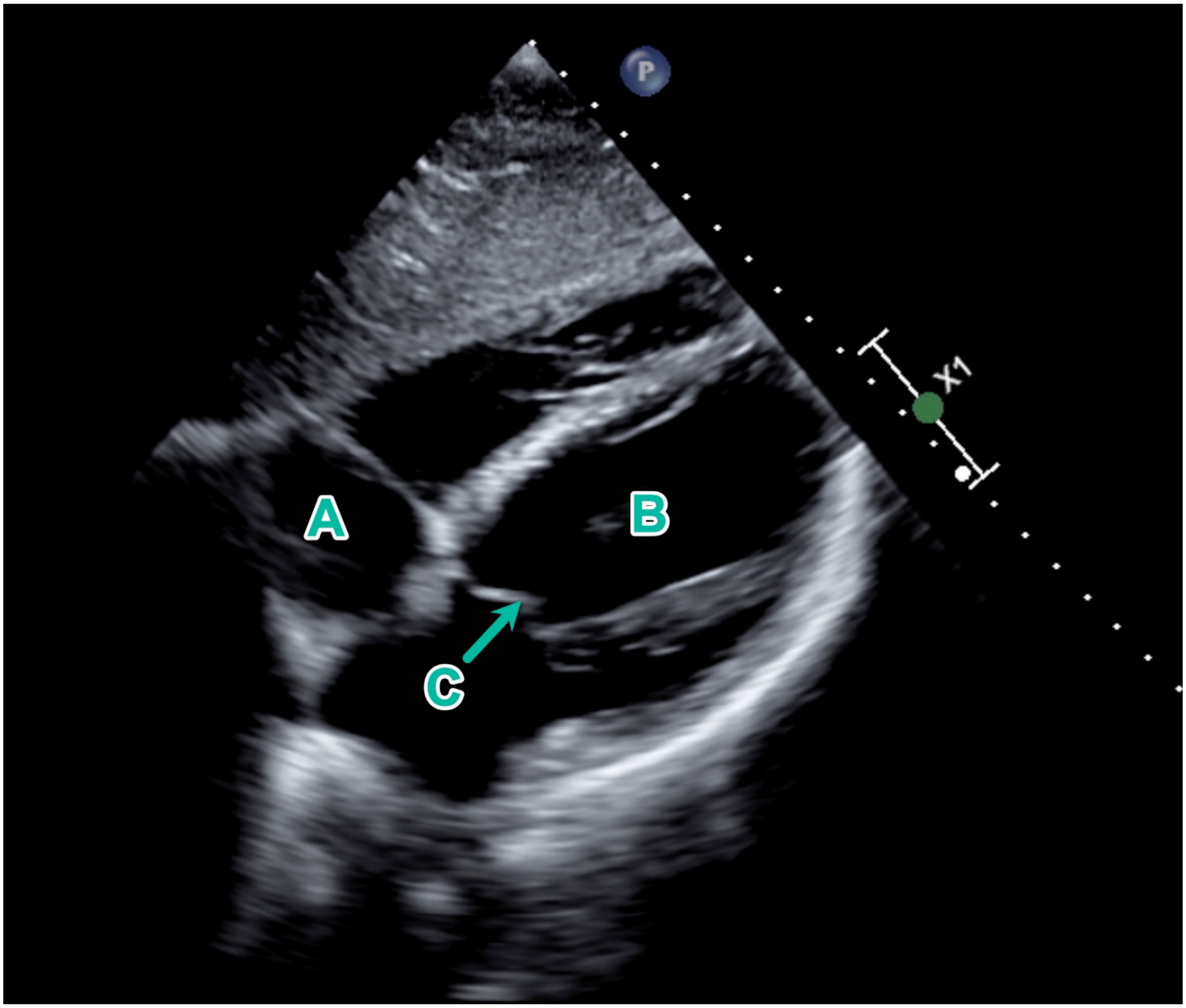

9) What axis of the heart is shown in the image? Answer: Long-axis.

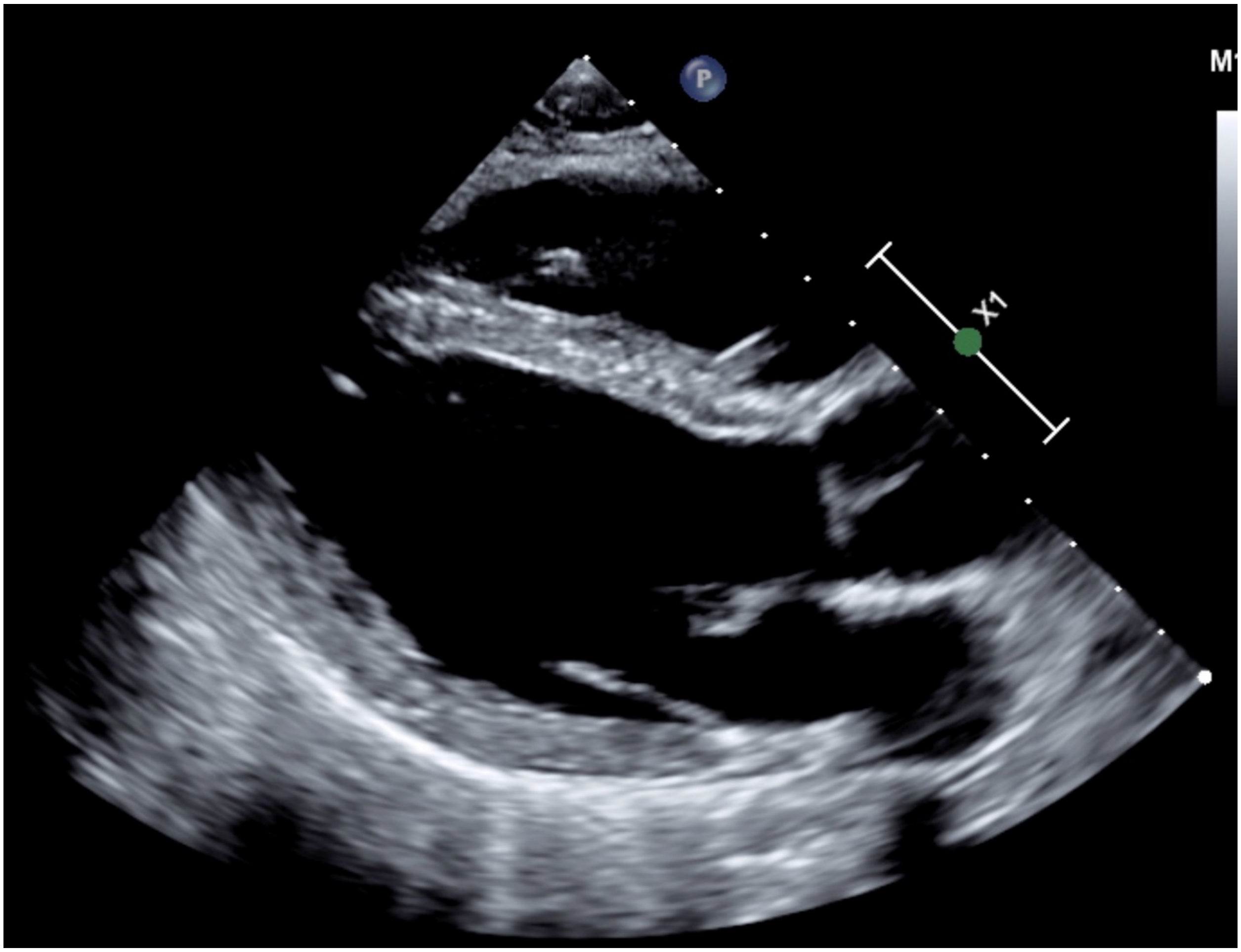

10) Identify the following structures (see image). Answer: A. Mitral valve. B. Right Ventricle.

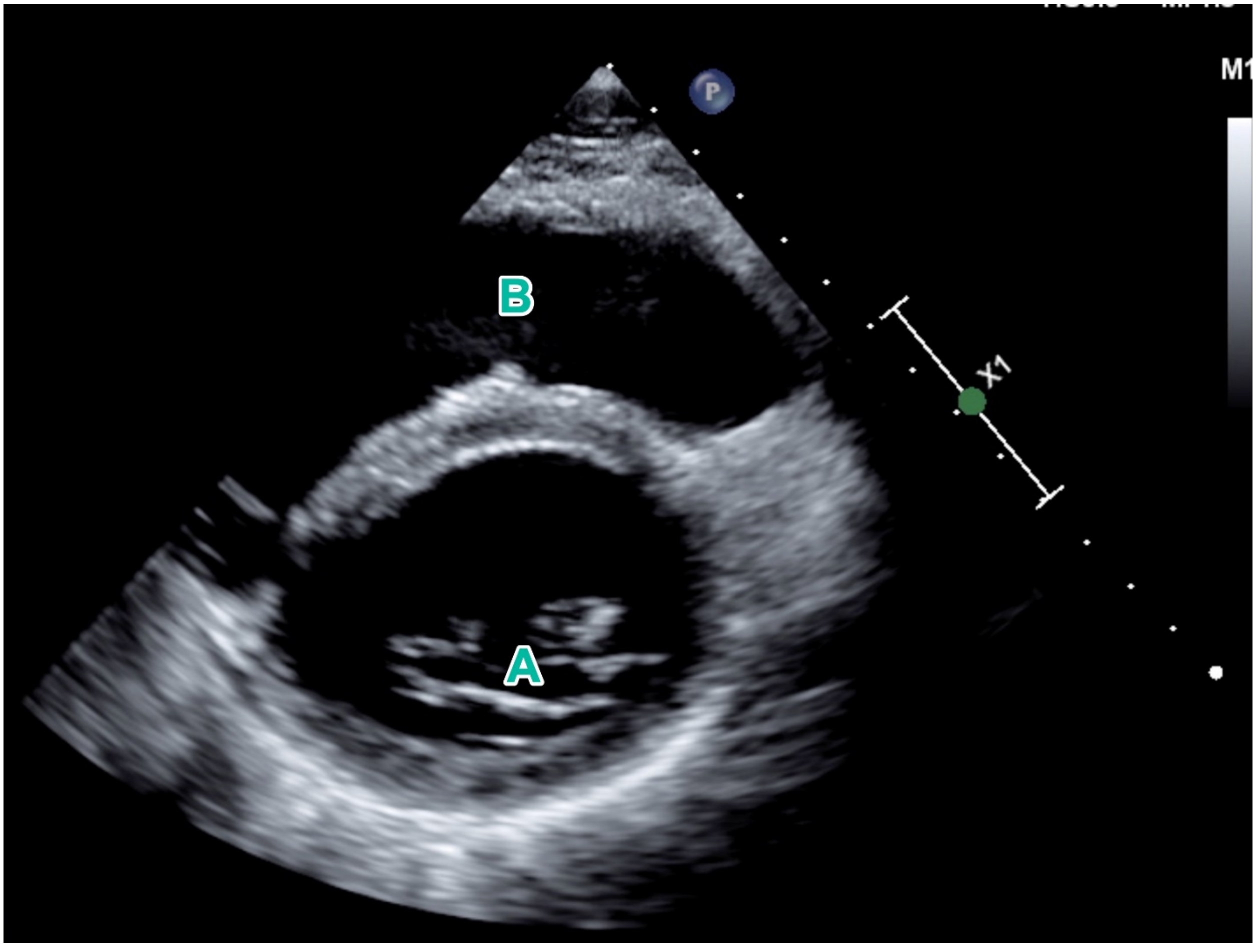

11) In order to visualize the papillary muscles, what transducer movement would be required from the current window (see image)? Answer: Caudal.

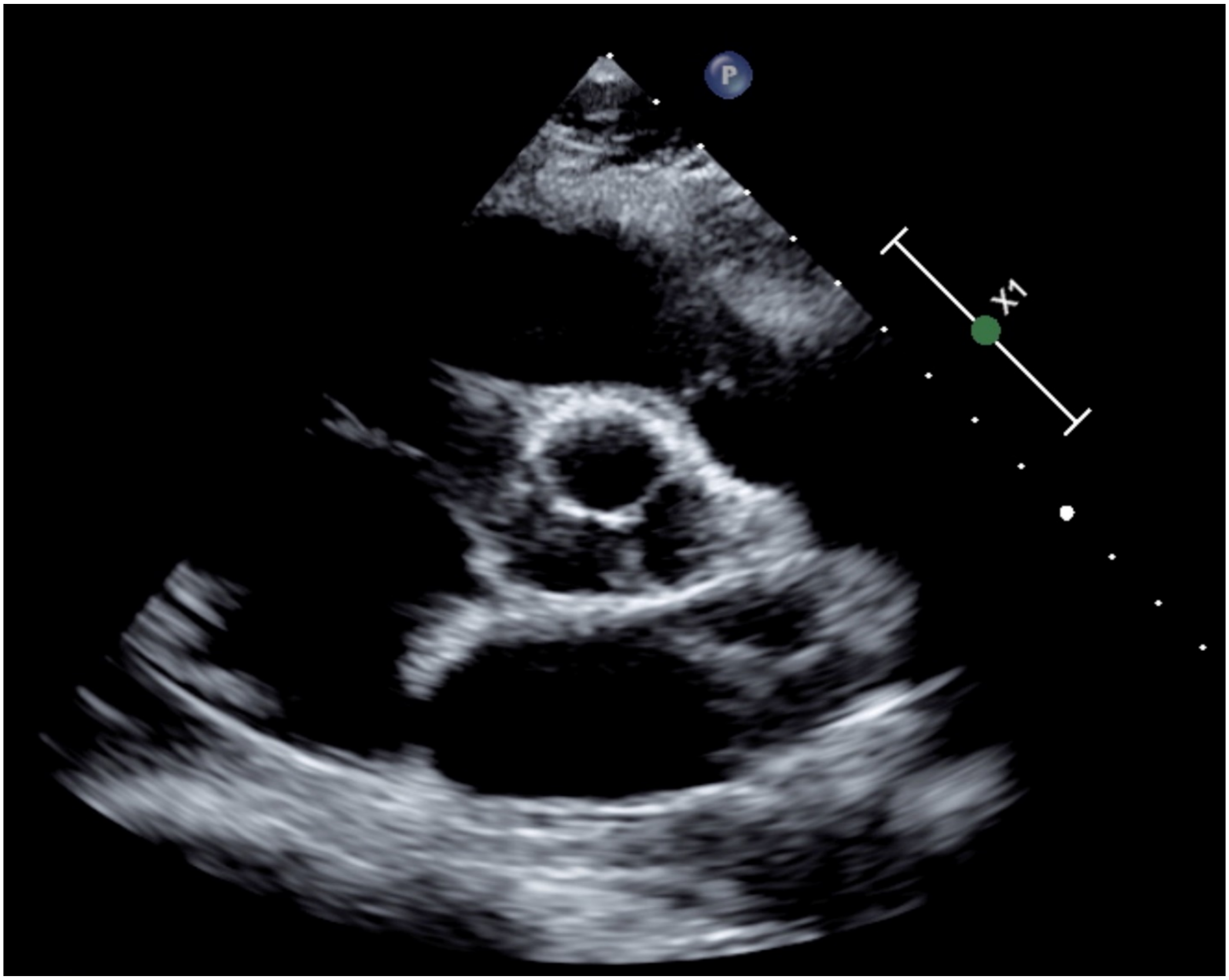

12) Identify the following structures (see image). Answer: A. Moderator Band. B. Papillary Muscle.

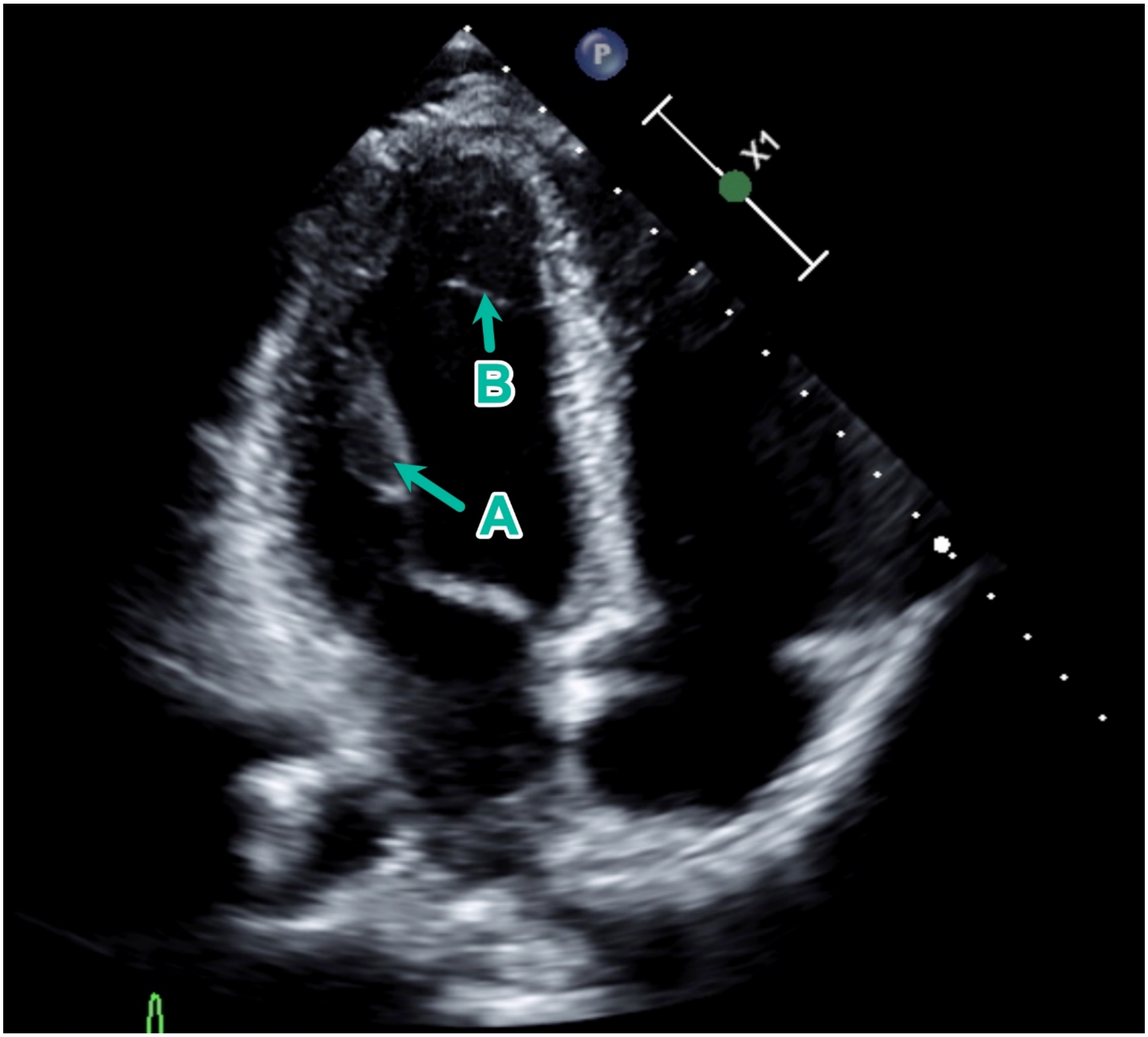

13) In order to visualize the aortic valve in short axis, what transducer movement would be required from the current window (see image)? Answer: 90-degrees clockwise rotation.

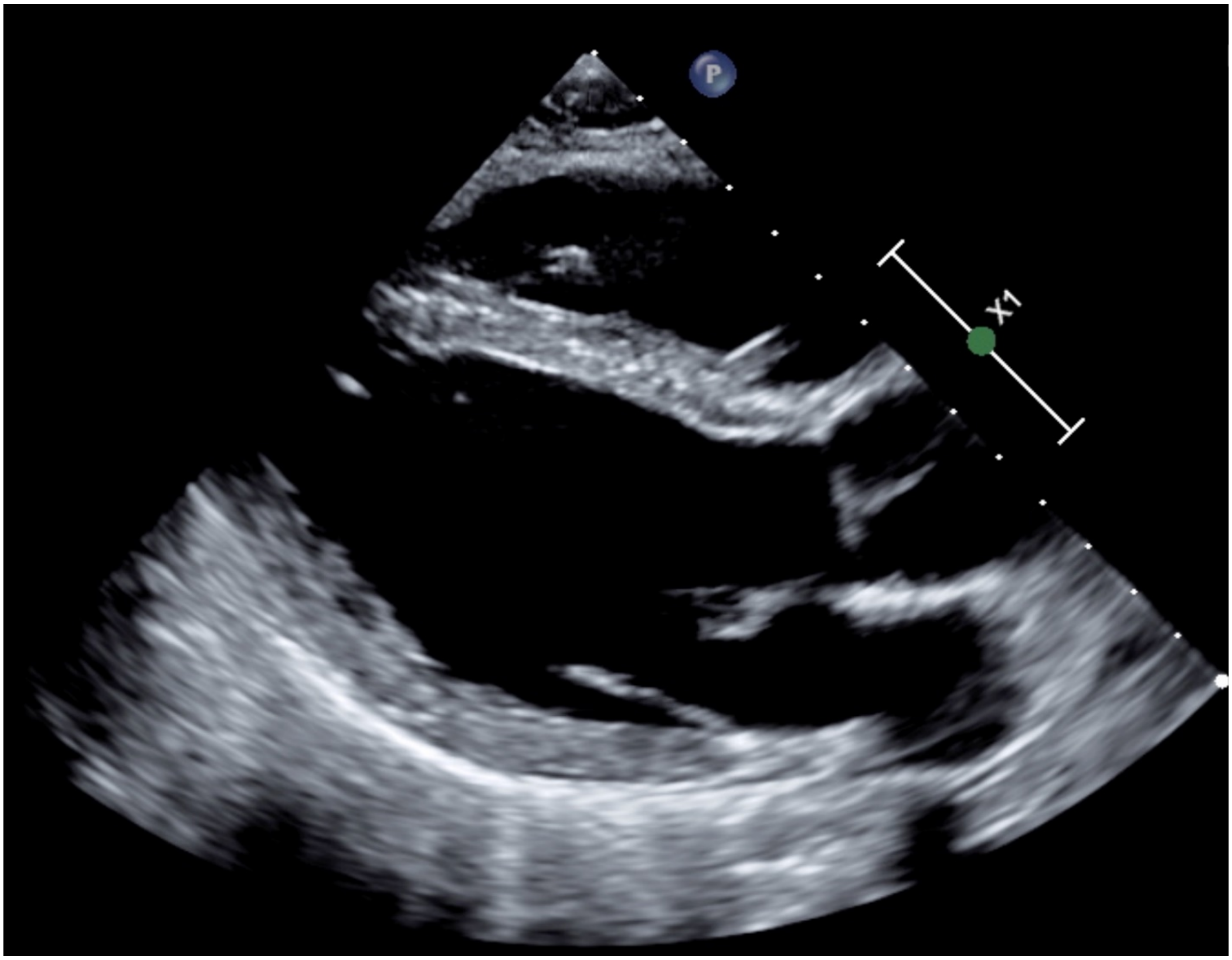

14) This image was obtained by placing the transducer in what anatomic location? Answer: Subcostal space.

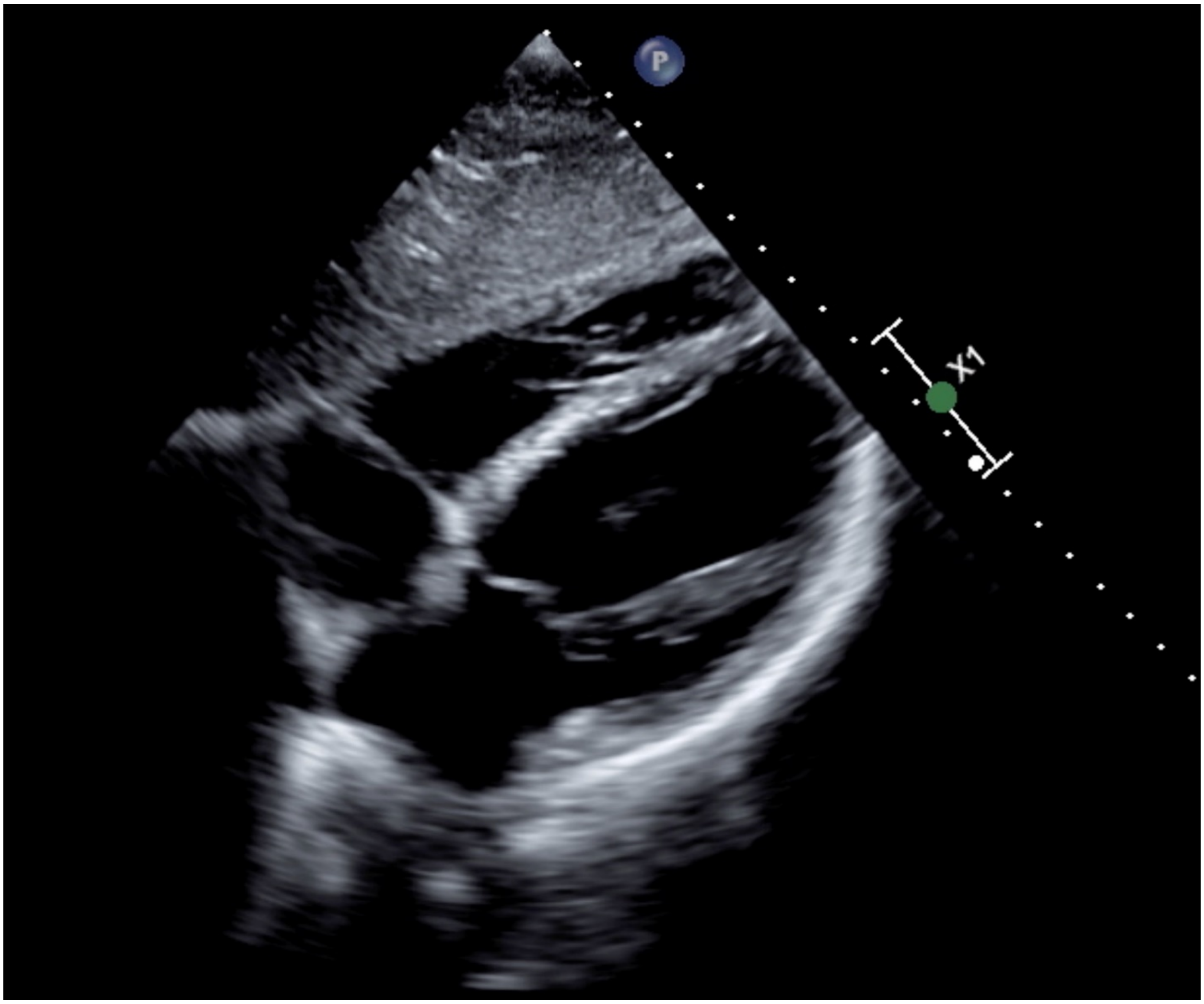

Supplement: Supplementary file 3 — Supplementary file3 (PDF 1404 KB) [file 40670_2025_2392_MOESM3_ESM.pdf]
