# Supplementary material for: Comparing the Effectiveness of Digital 3D PDF vs. 3D-Printed Heart Models as Learning Aids for Echocardiography in Medical Students
Source: Med Sci Educ. 2025 Apr 29;35(4):1983–92. doi: 10.1007/s40670-025-02392-x (PMC12532530; doi:10.1007/s40670-025-02392-x)

DELAYED POST-TEST KEY

6) Identify the following structures (see image). Answer: A Mitral valve. B. Tricuspid valve.

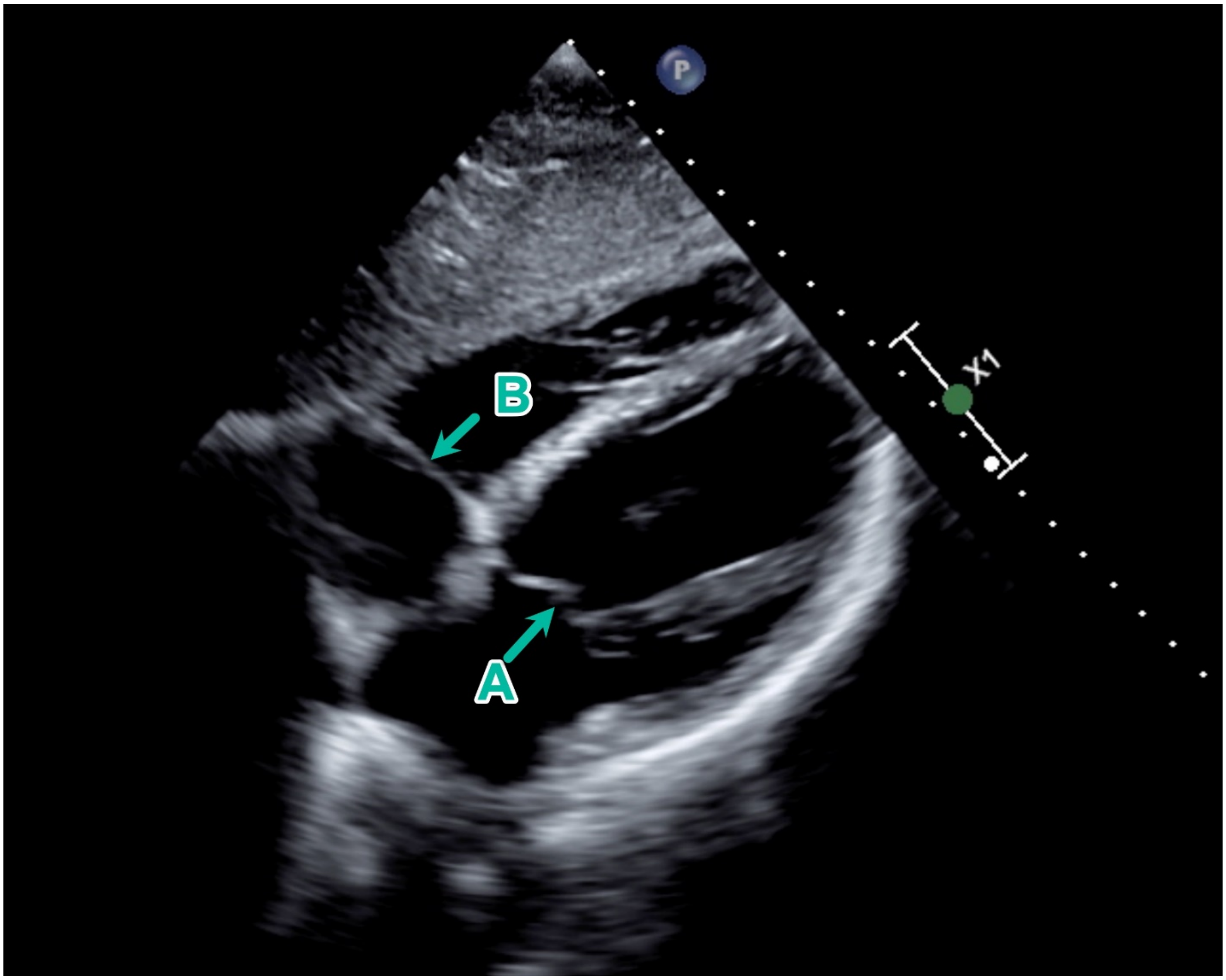

7) In order to visualize the aortic outflow tract, what transducer movement would be required from the current window (see image)? Answer: Angle beam anterior.

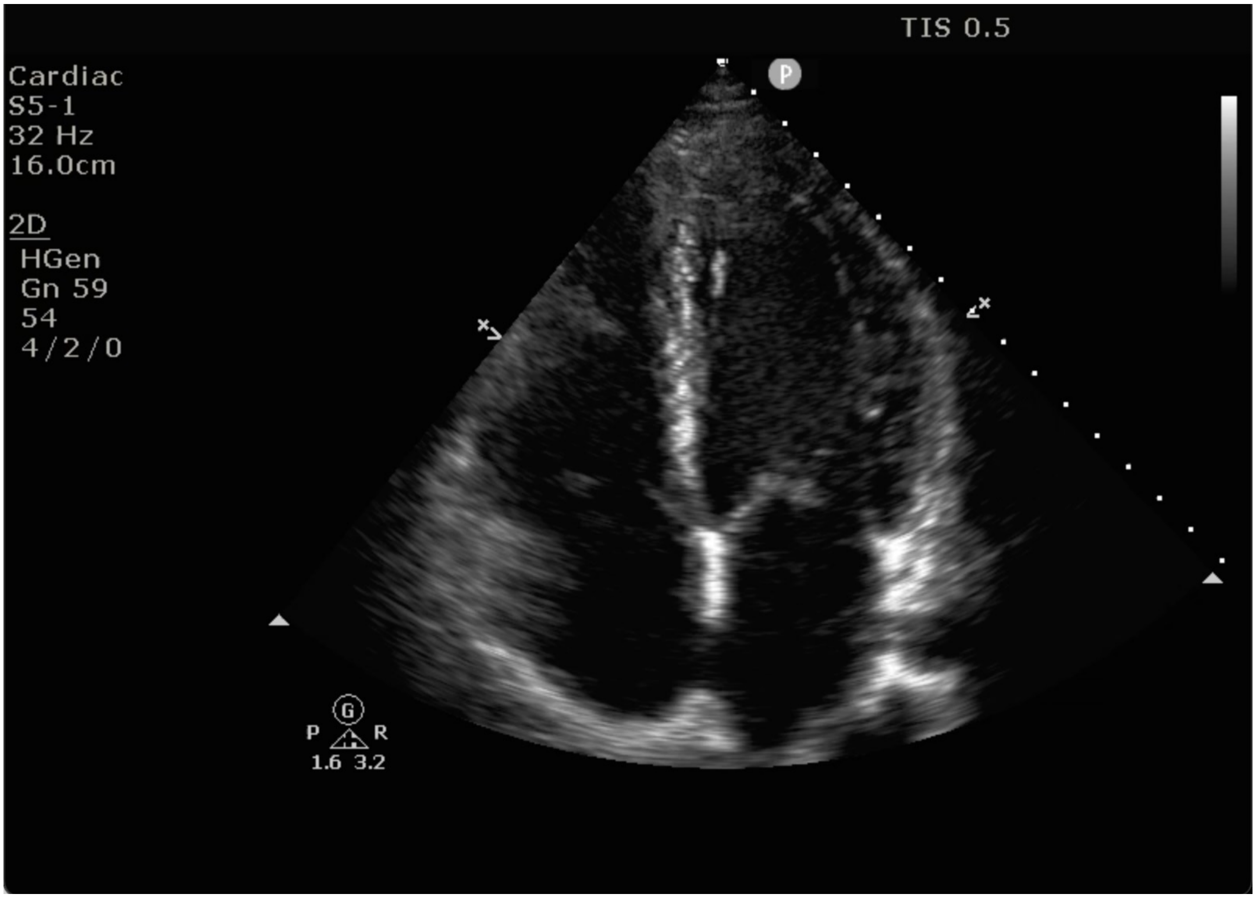

8) Identify the following structures (see image). Answer: A. Tricuspid valve. B. Left Atrium. C. Aorta or Aortic valve.

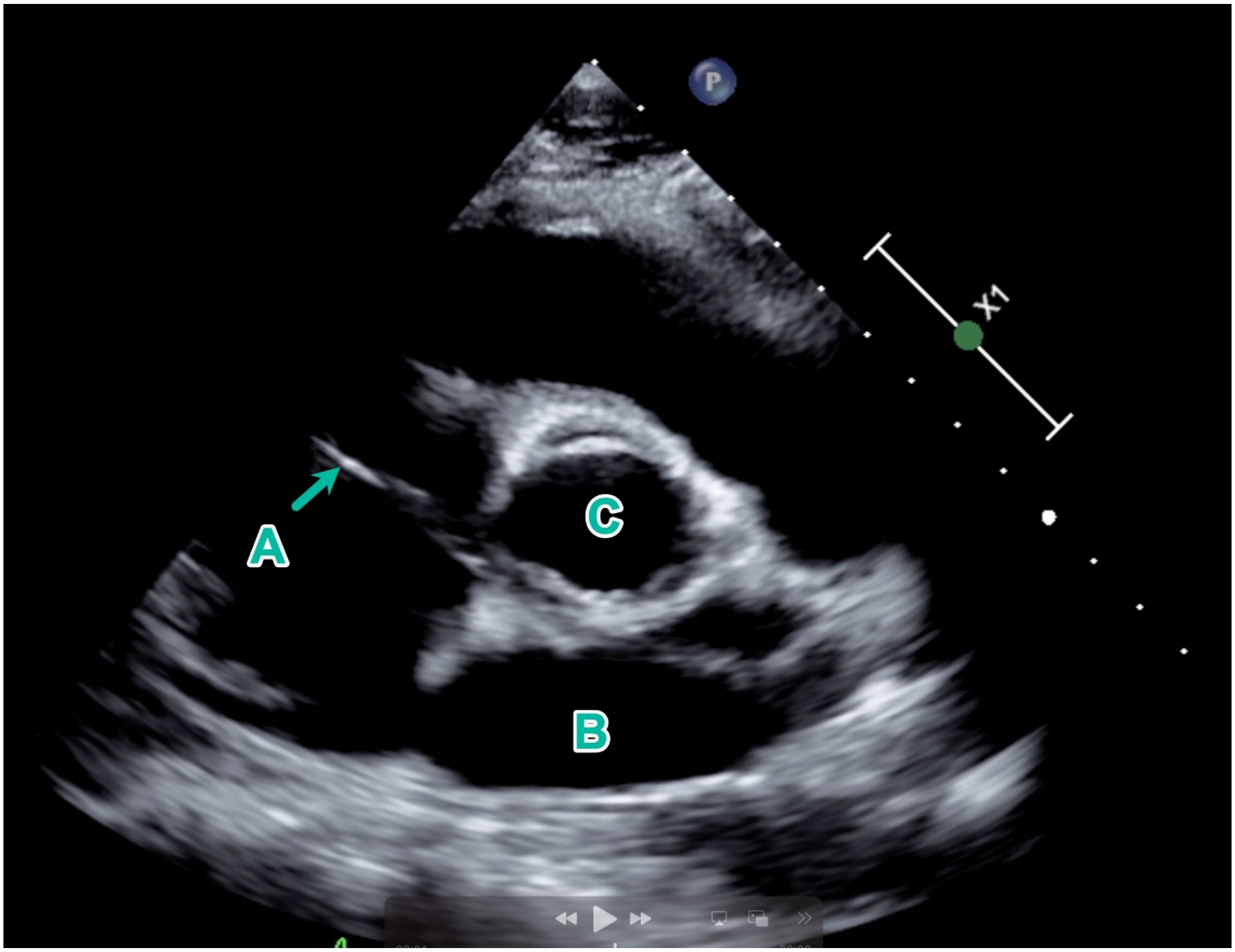

9) What axis of the heart is shown in the image? Answer: Long-axis.

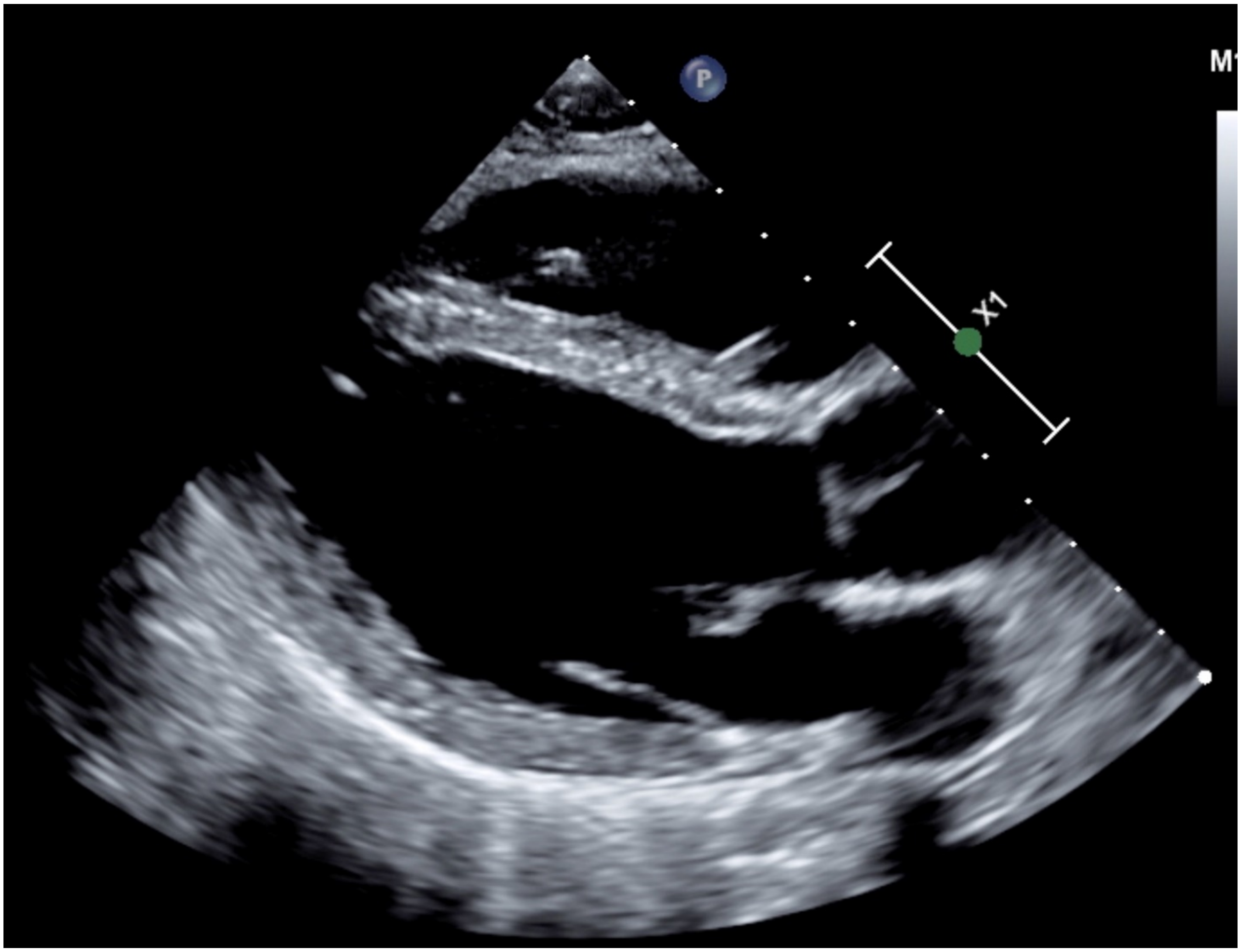

10) Identify the following structures (see image). Answer: A. Moderator Band. B. Papillary Muscle.

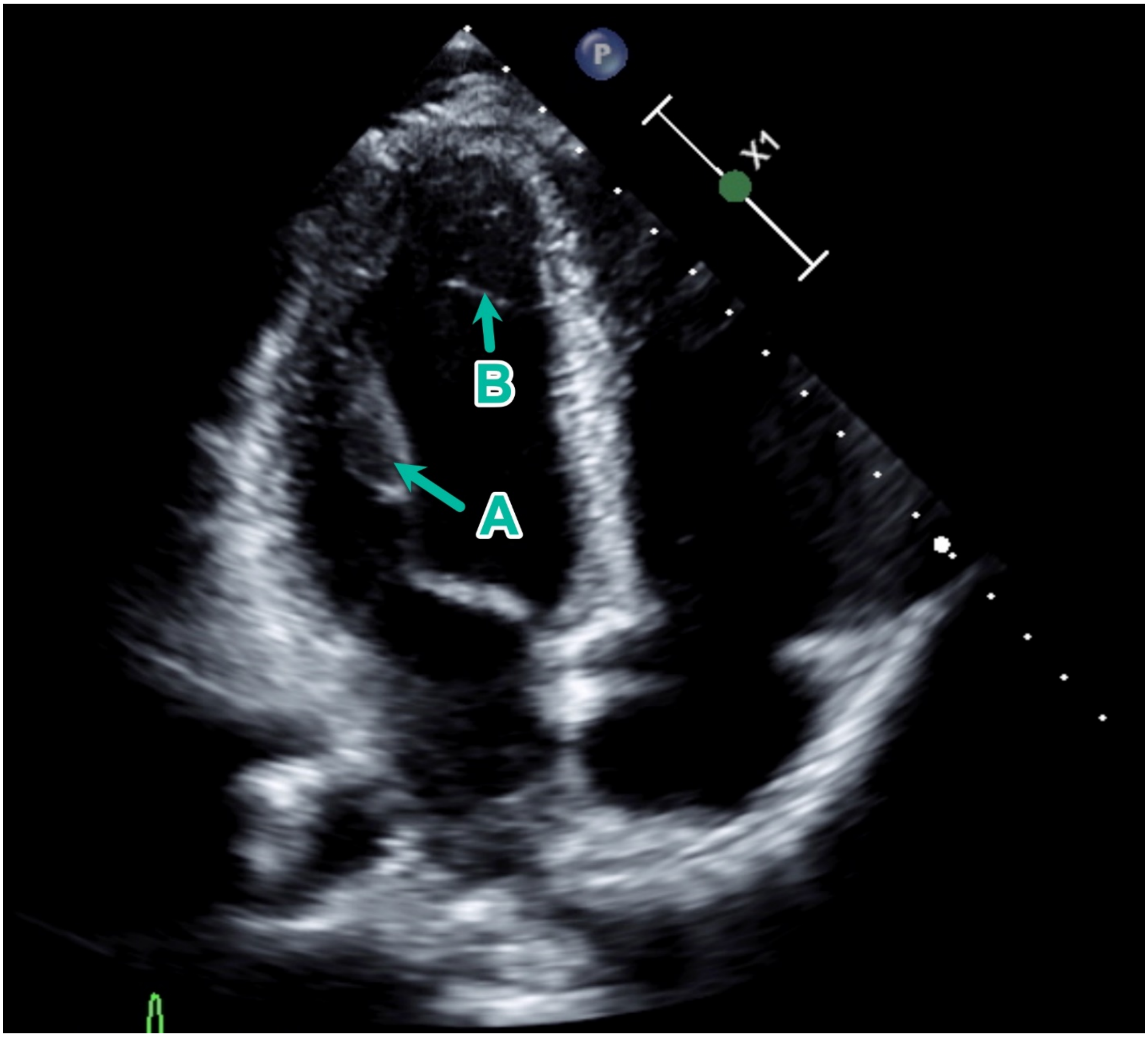

11) In order to visualize the aortic valve in short axis, what transducer movement would be required from the current window (see image)? Answer: 90-degrees clockwise rotation.

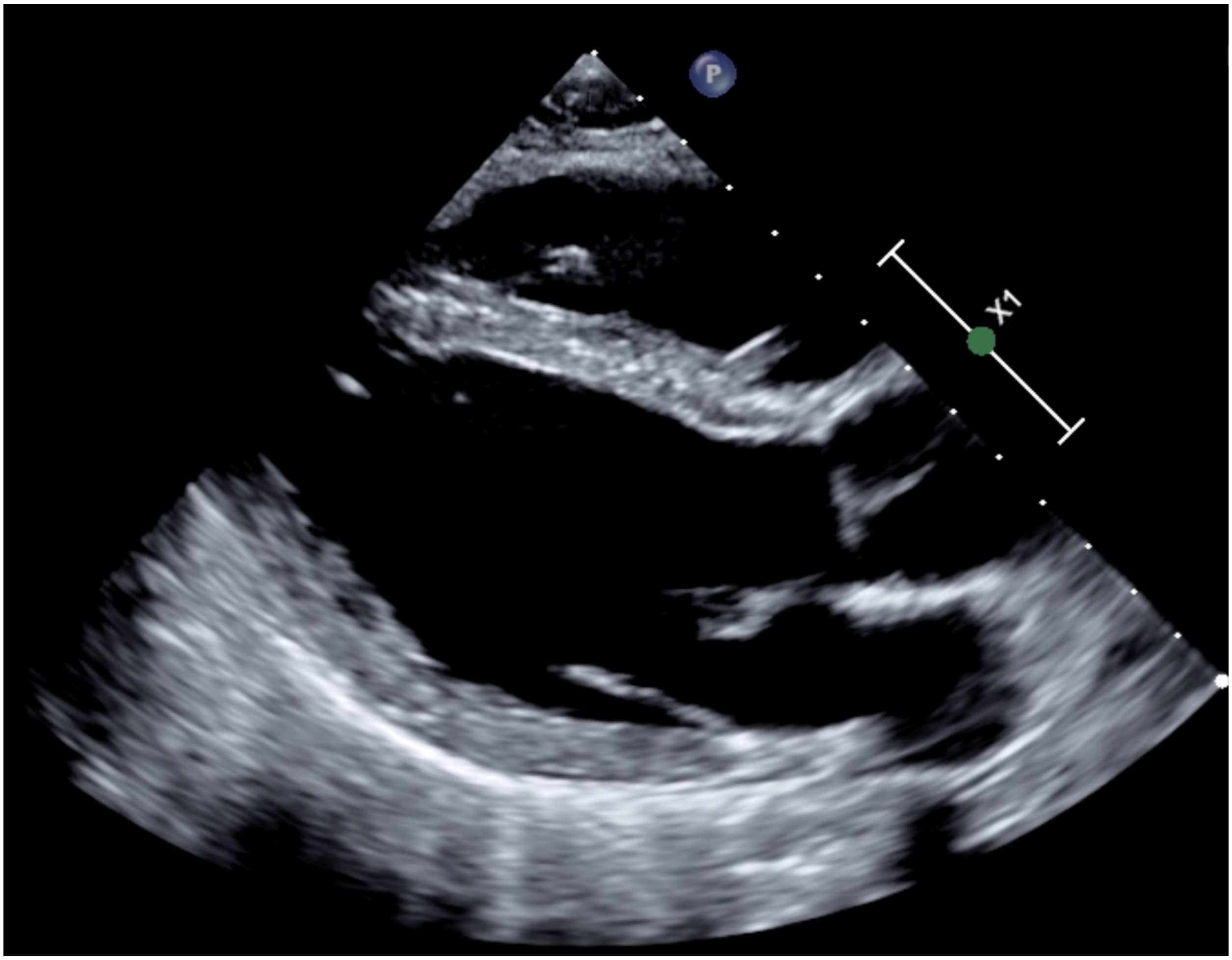

12) Identify the following structures (see image). Answer: A. Papillary Muscles. B. Right Ventricle.

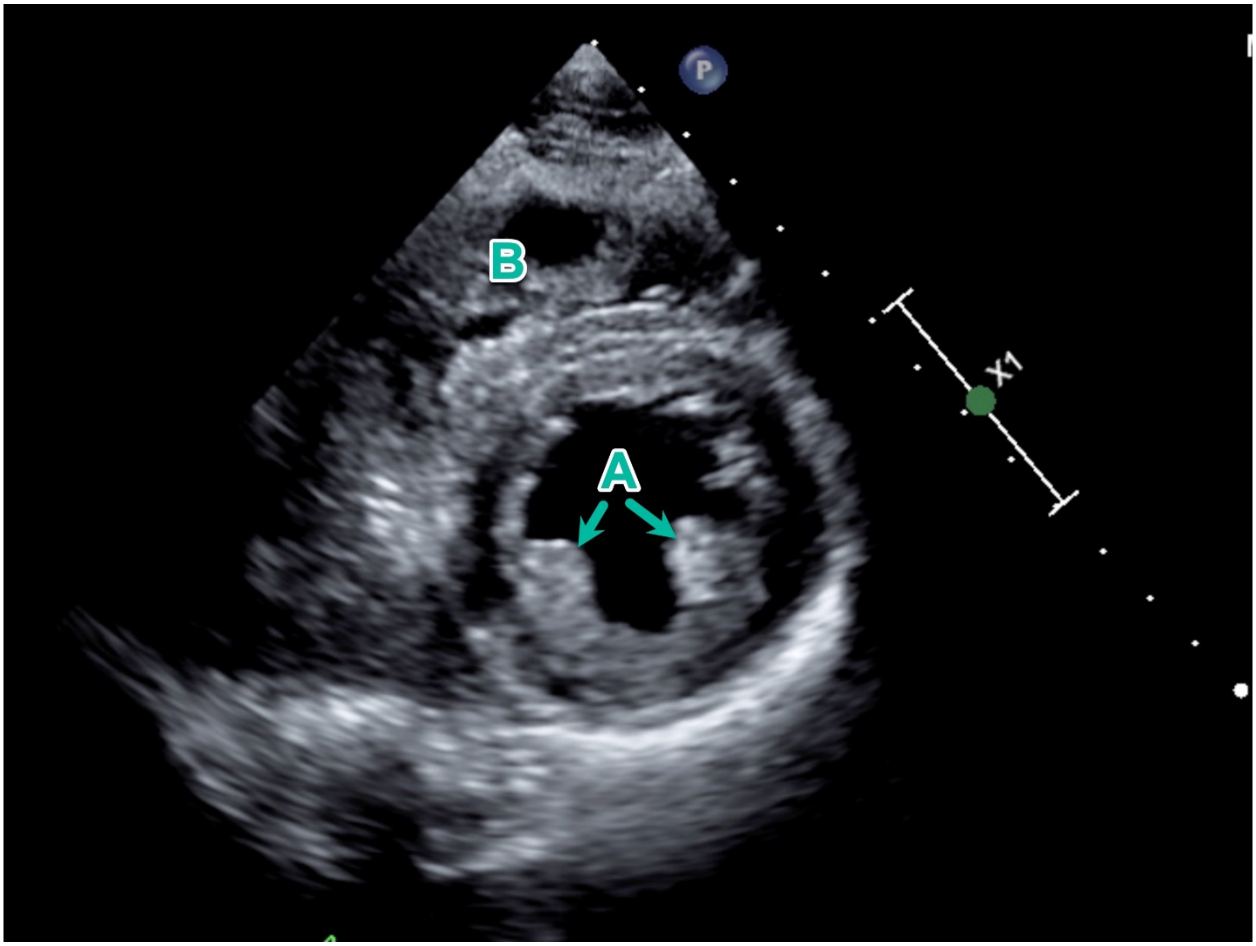

13) In order to obtain a window showing LV inflow and outflow, what transducer movement would be required from the current window? Answer: 90 degrees counterclockwise.

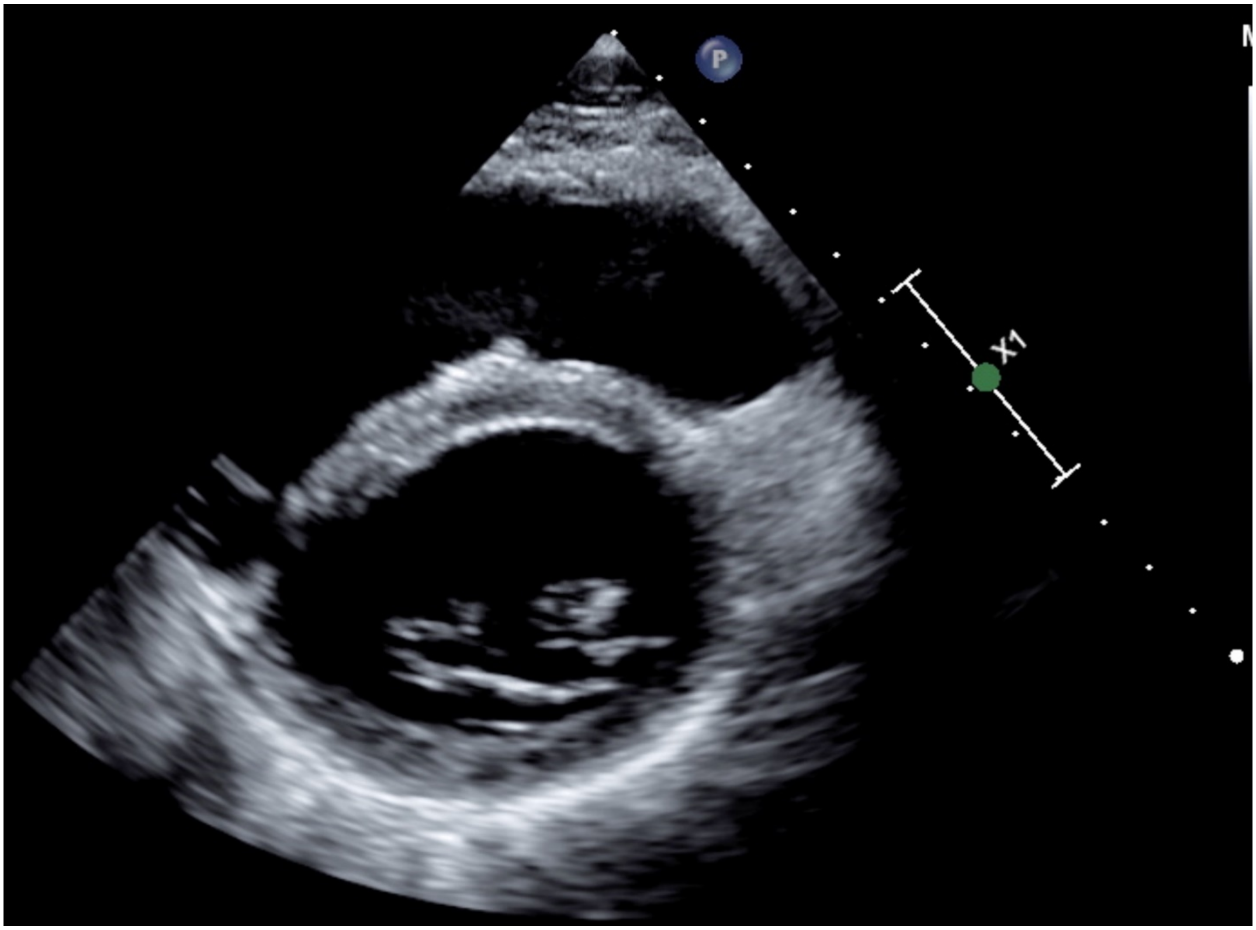

14) This image was obtained by placing the transducer in what anatomic location? Answer: Apical.

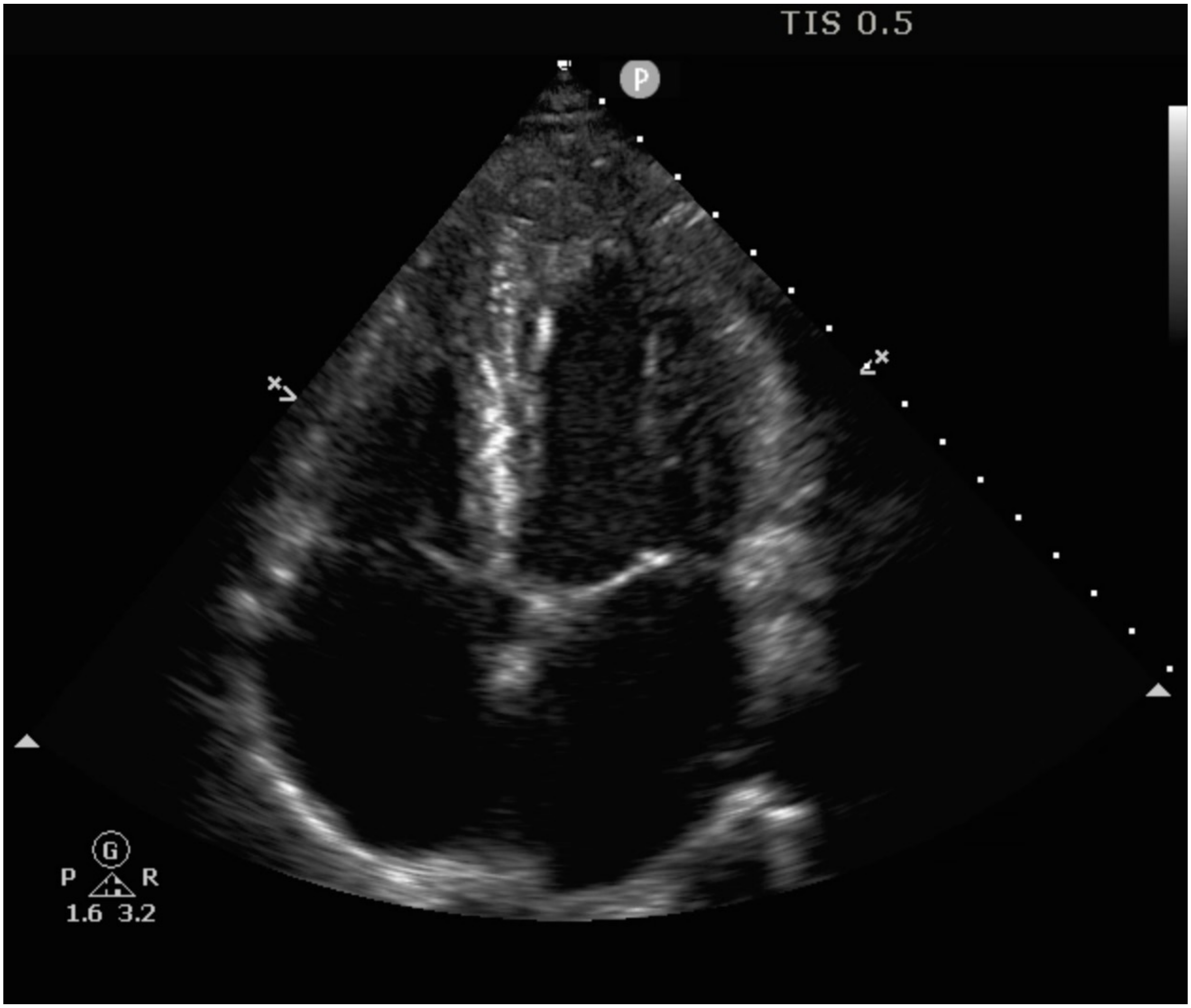

Supplement: Supplementary file 4 — Supplementary file4 (PDF 1310 KB) [file 40670_2025_2392_MOESM4_ESM.pdf]
